# Modeling Logical Interdependencies: Quantifying Systemic Risk From Rule‐Governed Actions

**DOI:** 10.1111/risa.70387

**Published:** 2026-10-14

**Authors:** Sukrati Gautam, David J. Yu, Hoon C. Shin, Mariam Alrefaei, Daniel Eisenberg, Changdeok Gim, Jeryang Park, Satish V. Ukkusuri

**Affiliations:** ^1^ Lyles School of Civil and Construction Engineering Purdue University West Lafayette Indiana USA; ^2^ Ecological Sciences and Engineering Purdue University West Lafayette Indiana USA; ^3^ Department of Political Science Purdue University West Lafayette Indiana USA; ^4^ Division of Humanities and Social Sciences Pohang University of Science and Technology (POSTECH) Pohang Republic of Korea; ^5^ Environmental Engineering and Sustainability Department College of Engineering and Energy Abdullah Al Salem University Kuwait City Kuwait; ^6^ Department of Operations Research Naval Postgraduate School Monterey California USA; ^7^ Department of Technology and Society State University of New York Incheon Republic of Korea; ^8^ Department of Civil & Environmental Engineering Hongik University Seoul Republic of Korea; ^9^ Department of Computer Science Purdue University West Lafayette Indiana USA

**Keywords:** Bayesian network, infrastructure resilience, institutional grammar, interdependencies in critical infrastructure, logical interdependencies, STAMP

## Abstract

Systemic risk in critical infrastructure is often analyzed through physical, geographic, and cyber interdependencies. Yet infrastructures are also interdependent through the governing rules that organize human action. These rule‐mediated connections are a form of logical interdependency, and breakdowns in rule‐governed action can create pathways through which disruption spreads. This paper develops a framework for diagnosing and quantifying this underrecognized source of systemic risk. It does so by modeling logical interdependencies as rule‐mediated control relations that shape whether interconnected infrastructures remain within safe operating bounds or drift toward failure. Integrating Institutional Grammar, STAMP, and Bayesian inference, the framework translates governing rules into structured control relations, represents them within a socio‐technical control architecture, and quantifies how failures in those relations propagate across interconnected infrastructures. We demonstrate the framework through a reservoir system case study centered on Lake Mendocino, California, where safety depends not only on reservoir infrastructure and hydrologic conditions, but also on distributed authority, communication requirements, and operational protocols. The results show that logical interdependencies make non‐trivial contributions to modeled systemic risk, with baseline probabilities of 7% for Coyote Valley Dam failure and 3% for downstream community impacts under the modeled conditions. More importantly, the analysis reveals *hidden logical risk*: rules and control relations that appear routine in normal operation yet become disproportionately important when the Bayesian model traces which factors most strongly contribute to a failure scenario. The paper moves logical interdependencies from a residual conceptual category to a systematically identifiable and quantitatively analyzable pathway of systemic risk.

## 1 Introduction

Cascading failures involving critical infrastructure, and the broader challenge of understanding and modeling systemic risk, are central concerns in risk analysis and resilience engineering (Chang et al. 2014; Cutter 2018; Pescaroli and Alexander 2016; Yu et al. 2020). Much of the existing work in this area has focused on technical linkages that are visible and readily formalized. Examples include material input‐output relations among infrastructures, such as the dependence of water systems on electric power, and inter‐system dependencies arising from spatial proximity or exchange relations (Ouyang 2014; Rinaldi et al. 2001). These kinds of linkages are central to the analysis of cascading disruptions because their visibility and formal tractability lend themselves to network representation, simulation, and scenario‐based modeling (Figure 1A). While this focus has generated important insights, we argue that it leaves the analysis incomplete.

**FIGURE 1:**
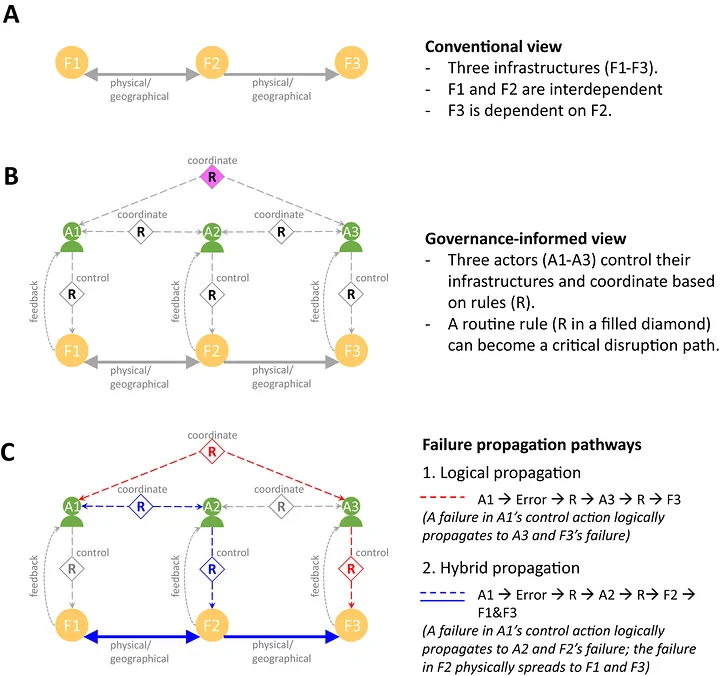
Conceptual contrast between conventional interdependency analysis and the proposed framework. (A) Conventional analysis emphasizes visible couplings among infrastructures. (B) The proposed framework treats infrastructures as governed systems in which actors control and coordinate operations through rules and protocols. A hidden critical rule, shown as R in a filled diamond, may appear routine under normal conditions but become a decisive pathway for cross‐system disruption when its execution breaks down. (C) Failure may then propagate through purely logical pathways or through hybrid pathways that combine rule‐mediated and physical interdependencies.

In many real‐world systems, disruptions do not propagate through these tangible channels alone. They also propagate through *logical interdependencies*: less tangible mechanisms rooted in human decisions that are not reducible to physical, geographic, or cyber connections (Rinaldi et al. 2001). More specifically, such decisions are often structured by governing rules and practices: operating procedures, reporting requirements, emergency protocols, and chains of authority that connect one organization's action to another system's operating condition (Yu et al. 2024). Although non‐physical, this configuration of rules, protocols, and authority relations—what we later call institutional arrangements (North 1990)—can become decisive under stress, when failures to follow a rule or protocol, such as a delayed notification, an omitted coordination step, or a misapplied protocol, alter how interconnected systems behave (Figure 1B,C). For risk analysis, the critical point is that systemic risk cannot be understood fully by tracing only material, spatial, and informational pathways; it must also account for the fact that infrastructures are *governed* systems, and that protocols structuring human action can *themselves* constitute pathways through which disruptions propagate, thereby generating systemic risk.

In this paper, we develop a diagnostic framework for analyzing and quantifying how institutional arrangements organize human action and contribute to systemic risk across interconnected infrastructures. Our aim is not simply to show that governing rules matter, but to explain when and how they become cross‐system failure pathways. More specifically, the paper is motivated by two questions. First, how can disruption propagation across critical infrastructures be analyzed, before failure occurs, as a process shaped jointly by human action and governing rules? Second, which rule‐governed actions and control relations appear routine under normal conditions, yet become decisive pathways of cross‐system disruption when errors occur in carrying them out?

This problem is not unique to any single event or infrastructure sector. Across interdependent infrastructure systems, disruption often depends not only on physical or cyber linkages, but also on the rules, procedures, and authority structures through which systems are prepared, operated, and coordinated. The 2021 Texas Winter Storm illustrates this broader problem. The event is often described as a case in which disruptions in electric power cascaded into water, healthcare, transportation, and other critical services (Busby et al. 2021; Tiedmann et al. 2023). That characterization is accurate, but incomplete. The severity of the crisis was shaped not only by dependencies among physical systems, but also by governing rules and practices: winterization was not required despite prior warning, and blackout procedures did not adequately protect some downstream services (Tiedmann et al. 2023). The case therefore illustrates a general analytical problem: without accounting for institutional arrangements, cross‐system disruption may be traced, but not fully explained. It also highlights the need for a framework that can analyze how governing rules shape disruption propagation across interdependent infrastructure systems.

The literature on infrastructure interdependency aligns with this view. Rinaldi et al.’s (2001) seminal work distinguished four principal ways in which infrastructures are interdependent: physical (arising through material flows), geographic (arising through spatial proximity), cyber (arising through information flows), and logical (arising through human actions). Logical interdependency was introduced to capture cases in which the state of one infrastructure depends on another through mechanisms that are not physical, cyber, or geographic, with human decisions often playing a central role. Yet logical interdependency remains the least clearly defined of the four categories. Because it was specified largely by exclusion rather than by a clear positive definition, it became a broad residual category encompassing many kinds of human influence, which made systematic analysis difficult (Yu et al. 2023).

More recent work by Yu et al. (2024) helped clarify this conceptual weakness by shifting attention from human decisions in the abstract to the formal and informal rules that specify what actors must do, may do, or must not do. Such rules allocate authority, prescribe actions, and organize coordination, thereby encoding relations among actors, actions, and objects. Their relational structure can be represented in network form, extending socio‐technical network approaches by making the rule layer explicit (e.g., Manny et al. 2022; Olivier et al. 2025; Yu et al. 2024). This makes rule‐mediated logical interdependencies more systematically identifiable and analytically tractable. The same rule‐focused approach also clarifies that logical interdependencies rarely exist in isolation (Gim and Miller 2022; Yu et al. 2024): in real systems, they often coexist with physical, geographic, or cyber connections, producing hybrid forms of interdependence (Figure 1C). For example, underground water pipes and road networks may be geographically interdependent because of spatial proximity, while also being linked through rules and procedures governing maintenance and repair work near adjacent systems.

A complementary quantitative literature uses agent‐based and system‐of‐systems models to represent human decisions and interactions within infrastructure systems (e.g., Eusgeld et al. 2011; Nan and Sansavini 2016; Sun et al. 2019). These approaches are particularly useful for representing dynamic interactions, adaptation, and emergent behavior among infrastructure operators, organizations, and affected communities. In such models, however, institutional influences on decisions are generally represented through model‐specified behavioral rules or decision mechanisms, rather than systematically derived from governing rule text itself.

These two lines of work address different parts of the problem but leave an important gap between them. Rule‐focused studies provide a stronger basis for identifying how actual governing rules structure relations among actors, actions, and infrastructures, but have not yet shown how failures in those relations propagate across interconnected systems, including through hybrid pathways in which logical interdependencies interact with physical, geographic, or cyber connections. Agent‐based and system‐of‐systems models, meanwhile, provide powerful tools for representing these processes, but generally do not treat the content and structure of governing rule text itself as the starting point of the analysis. What remains missing is a framework that connects these capabilities: one that begins from actual governing rules, traces how failures in rule‐governed actions propagate across interconnected infrastructures, and evaluates these pathways in risk terms, including which rules and control relations matter most for system safety and which become especially consequential when disruption occurs.

Meanwhile, the accident and system‐safety literature shows a similar conceptual shift. Earlier accident models, along with many conventional risk‐analysis tools, generally treated failures as linear sequences of events or as malfunctions of identifiable components (Patriarca et al. 2022; Zhang et al. 2023). Such approaches remain useful, but are less well suited to systems in which safety depends on multilevel coordination, distributed authority, delayed feedback, and nonlinear interaction. Rasmussen's AcciMap (Rasmussen 1997) marked an important advance by incorporating laws, regulation, management, and organizational processes into accident analysis. Yet even these event‐centered approaches are less able to represent governing rules and control constraints that do not operate as simple chains of events.

Leveson (2004)’s Systems‐Theoretic Accident Model and Processes (STAMP) offers a more suitable foundation for this problem. STAMP treats safety not primarily as the prevention of component failure, but as a problem of control. In this view, socio‐technical systems are maintained within safe bounds through control structures such as safety constraints, feedback processes, and control actions. Accidents occur when those constraints are absent, inadequately designed, poorly communicated, not enforced, or undermined by dysfunctional interactions among components and actors. This perspective is especially useful here because governing rules, policies, and procedures can be understood as constraints embedded within a control structure rather than as external background conditions. STAMP thus provides an analytic language for bringing institutional arrangements into risk analysis. More broadly, it supports a view of accidents in which failure may arise not only from broken parts, but also from inadequate control and dysfunctional rule‐mediated interactions among otherwise functioning elements of a socio‐technical system.

However, STAMP by itself does not fully resolve the problem addressed here. Three limitations are especially relevant. Most STAMP applications have focused on individual systems (e.g., Ishimatsu et al. 2014; Rokseth et al. 2019; Shin et al. 2021; Wu and Huang 2019) rather than on multiple infrastructures linked through governing rules, overlapping authority, and cross‐organizational coordination. In addition, while STAMP can represent rules and policies as constraints within a control structure, it does not provide a systematic way to formalize governing rules so that cross‐infrastructure connections can be represented explicitly. Finally, STAMP remains largely qualitative, which limits its usefulness for comparing, prioritizing, and quantifying multiple rule‐mediated pathways. Recent efforts to integrate STAMP with Bayesian networks (BNs) address part of this limitation by translating STAMP‐derived hazards, unsafe control actions, and contributing factors into Bayesian structures that support probabilistic inference and sensitivity analysis (Meng et al. 2022; Ozan Ceylan et al. 2025). Yet these studies have largely remained within single‐system domains. They show how a STAMP‐informed control structure can be quantified, but they do not yet provide a framework for representing how rule‐based connections spanning multiple infrastructures become pathways through which disruptions propagate and systemic risk is generated. The remaining challenge is to bring these strands together for the analysis of cross‐infrastructure systemic risk.

The objective of this paper is to develop a diagnostic framework for analyzing how disruptions propagate across interconnected infrastructures through rule‐governed actions and control relations that existing approaches do not represent well, and how such propagation contributes to systemic risk. Situated at the intersection of infrastructure interdependency studies and systems‐oriented risk assessment, the framework combines a rule‐based understanding of logical and hybrid interdependencies with a control‐theoretic and quantitative approach to systemic risk analysis. More specifically, it uses Institutional Grammar to translate governing rules into analyzable inputs (Crawford and Ostrom 1995; Siddiki et al. 2019), extends STAMP (Leveson 2011) to represent how rule‐mediated relations shape cross‐system failure pathways through control structures and inappropriate control actions (ICAs), and incorporates Bayesian inference to quantify the systemic risk associated with those pathways. The paper thus advances a method for analyzing the systemic consequences of rule‐mediated connections. This risk‐analysis perspective also complements resilience approaches that examine how systems maintain, adapt, and recover function under disruption (Sun et al. 2019; Yu et al. 2020). By connecting governing rules to control failures and modeled consequences, the framework can assess whether governance arrangements fit the infrastructure dependencies they are meant to manage and where that fit may be weak, including gaps in the information exchange, coordination, and authority needed to manage those dependencies. Such diagnoses can inform changes that strengthen the collective capacity to respond and adapt under stress—capacities that are central to resilience—although the framework does not directly measure resilience gains.

We demonstrate the utility of this framework through a reservoir case centered on Lake Mendocino, California. This setting is well suited because reservoir safety depends not only on physical infrastructure and hydrologic conditions, but also on layered governance arrangements involving release schedules, communication requirements, and multiple levels of authority. In the case study, operational rules are extracted from a reservoir control manual and related governance texts, coded into analyzable rule components, and analyzed alongside physical and organizational components of the system, including linked dams and downstream communities. This allows the framework to represent not only rule‐mediated logical interdependencies, but also hybrid forms in which governing rules interact with physical, geographic, and organizational relations across infrastructures. The case shows how seemingly routine governing rules can create cross‐system disruption pathways with significant systemic risk implications.

## 2 Framework and Case Study

### 2.1 System Boundary and Rule Corpus

This section presents the diagnostic framework through its implementation in the Lake Mendocino reservoir system in northern California (Gautam et al. 2025). For the purposes of the analysis, the Lake Mendocino system is represented as three broad but interdependent infrastructure groups (Figure 2A). The first is an upstream group centered on Potter Valley Powerhouse Diversion Tunnel and related inflow conditions. The second is a core reservoir group centered on Lake Mendocino and Coyote Valley Dam. The third is a downstream group that includes municipal water users, irrigation systems, recreational functions, and communities potentially affected by releases or operational failure. This three‐part representation is not intended as an exhaustive model of the full Russian River basin. Instead, it defines a boundary broad enough to capture the cross‐system consequences of operational decisions while remaining analytically tractable for rule coding, STAMP‐based analysis, and probabilistic modeling.

**FIGURE 2:**
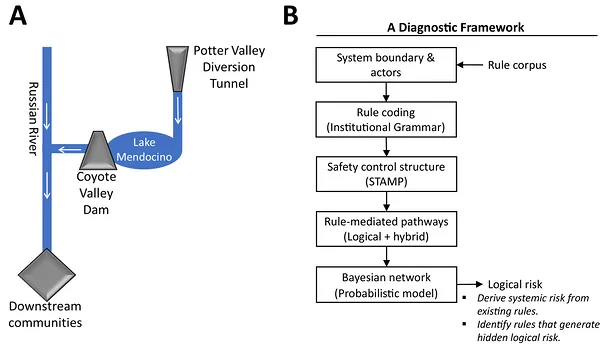
(A) High‐level schematic of the three main infrastructure groups in the Lake Mendocino system used in the multi‐infrastructure interdependency analysis: upstream infrastructure (Potter Valley Powerhouse Diversion Tunnel), core reservoir infrastructure (Lake Mendocino Reservoir and Coyote Valley Dam), and downstream infrastructure and community systems (recreational facilities, irrigation, and communities). (B) Overview of the proposed diagnostic framework, which transforms a rule corpus into a logical risk model through successive stages of rule coding, safety control structure analysis, logical and hybrid pathway identification, and Bayesian network modeling and inferences.

The analytical scope is defined not only by the infrastructures included but also by the corpus of governing rules brought into the model. The relevant rules are drawn exclusively from the reservoir control manual *Coyote Valley Dam Control Manual* and its appendices (USACE 1986) which govern day‐to‐day operations, communication requirements, release scheduling, control actions, operating modes, and institutional authority over dam operation. Because the manual is extensive, the subset of rules presented in Table 1 is deliberately scoped to operational rules that involve more than one infrastructure or require coordination across infrastructures. The rule corpus therefore serves as the institutional counterpart to the physical boundary.

**TABLE 1:** A Set of rules for governing water release operations at Lake Mendocino.

Rule no.	Rule statements
R1	All water releases from Coyote Dam will be determined, controlled, and directed by the Corps.
R2	The Corps will establish, and transmit to the City, dam flow release schedules (except during flood control operation) at the Coyote Dam for a weekly, daily, or other period as needed, in units of cubic feet per second.
R3	The City, after receiving the flow release schedules, shall provide an operations program for that period.
R4	The operation program will include the flow distribution through all discharge valves and the power plant necessary to comply with the Corps release schedules.
R5	The Corps will be notified immediately of any revisions to this program.
R6	In no case will such revisions affect the Corps release schedules.
R7	The dam release schedules: operations program and revisions to the program will be transmitted by telephone or radio. Radio communication will be between the city and the Corps located in Sacramento, California on the City's supplied equipment and frequency.
R8A	(c) During emergency situations, as determined by the Corps, the Corps will advise the city of the situation
R8B	and may take appropriate action, including shutting down the power units and assuming control of the appropriate outlet gates and valves.
R9	Plant operation will be monitored 24 h per day.
R10	(f) The city will be responsible for maintaining a minimum dissolved oxygen level of 7.0 mg/L at the existing U.S.G.S. stream gage immediately downstream of the proposed tailrace.
R11	Power generation mode will be in operation when the required dam releases are between 0 and 4375 cfs, inclusive and the flood pool is at or below elevation 755 ft.
R12	Flood control mode will be in operation when the required dam releases exceed 4375 cfs, the flood pool exceeds elevation 755 ft, or the conservation pool exceeds elevation 762 ft.
R13	(h) When the dam and power plant facilities are operating in the power generation mode, the control gates (three service slide gates and three emergency slide gates in the intake tower) will be fully open, the plenun tainter valve will be fully closed, and the turbines and bypass valves will be opened as necessary to pass the required water release.
R14	(i) When the dam and power plant facilities are operating in the flood control mode the plenum tainter valve will be fully open, the turbines and bypass valves will be fully closed, and the control gates will be opened as necessary to pass the required water release.
R15	Sonoma County Water Agency (SCWA) diverts municipal water at its pumping stations in the vicinity of Wohler Bridge below Healdsburg.
R16	Pacific Gas and Electric Co. (PG&E) divert majority of the inflow water to the Lake Mendocino via the Potter Valley powerhouse diversion tunnel.
R17	Coyote Valley operators will provide routine and emergency instructions and support for Water Control Data Systems (WCDS).
R18	The city strongly supports the maintenance of maximum summer flows in the Russian River to protect water quality and the recreational values of the Russian River.

### 2.2 Overview of the Framework

Figure 2B summarizes the framework. The analysis proceeds in five linked stages. First, the socio‐technical system is delimited by identifying the relevant infrastructures, actors, and governance arrangements. Second, relevant rule texts are coded using institutional grammar into syntactic elements that make their relational structure explicit. Third, those coded rules are translated into a STAMP‐based representation of the safety control structure (SCS), ICAs, and causal factors. Fourth, logical and hybrid pathways of disruption identified from the STAMP model are used to construct a BN that formalizes their conditional structure. Fifth, the Bayesian model is parameterized using a combination of human‐reliability estimates and infrastructure failure rates so that systemic risk can be analyzed through baseline probability propagation and backward inference.

The five stages form a single analytic pipeline rather than a juxtaposition of separate methods. Rule coding converts textual rules into structured relational inputs. STAMP situates those inputs within a socio‐technical control architecture and identifies where deviations in rule‐governed action may generate hazardous conditions. Bayesian modeling then quantifies how those deviations can propagate across the system and contribute to systemic risk.

The remainder of this section explains the pipeline through the Lake Mendocino case. The framework treats formal rules as one analytically tractable layer of a broader socio‐technical system and examines how failures in that layer become consequential through interaction with physical infrastructure, communication interfaces, and operational thresholds. In this way, logical interdependencies become systematically identifiable and quantitatively analyzable contributors to systemic risk.

### 2.3 Rule Coding to Formalize Control and Coordination Relations

The first substantive step is to code the system's rule texts into structured elements that can be used analytically. For this purpose, the framework uses Institutional Grammar, a method from the broader institutional analysis tradition that decomposes rules into six syntactic elements: ATTRIBUTE, DEONTIC, AIM, OBJECT, CONTEXT, and OR‐ELSE (Siddiki et al. 2019). This step addresses a basic methodological problem. The rules governing infrastructure systems are written in legal and operational prose, not in a form that can be represented directly in a SCS or probabilistic network. Institutional grammar provides a disciplined way to move from text‐heavy directives to a relational representation that specifies who is expected to act, what action is required or permitted, which object is affected, and under what conditions the action applies.

In the present application, the systematic corpus‐level coding focused on four elements: ATTRIBUTE, DEONTIC, AIM, and OBJECT (Table A1 in the Supporting Information). ATTRIBUTE identifies the actor responsible for action, usually an agency or operating entity. DEONTIC specifies normative force, such as whether an action is required, permitted, or prohibited. AIM identifies the action itself. OBJECT specifies the recipient or target of that action, which may be another actor, a piece of information, or a physical infrastructure component. Contextual conditions were retained from the original rule text and used in interpreting when and how a rule applies during the subsequent control analysis, but CONTEXT and OR‐ELSE were not systematically tabulated across the full rule corpus. Box B1 in the Supporting Information illustrates how contextual information can nevertheless be identified from individual rule statements. Through this coding, prose directives are converted into structured elements that can be carried into the later stages of the framework. For example, Figure 3 shows how a rule statement *“(R1) Operator must do safety inspection and imp*rov*e debris collection facility, stormwater storage facility and discharge facility in preparation for stormwater overflows that often occur in times of monsoon or typhoon”* can be coded to extract rule components and their relational structure (see Box B1 in the Supporting Information for more examples).

**FIGURE 3:**
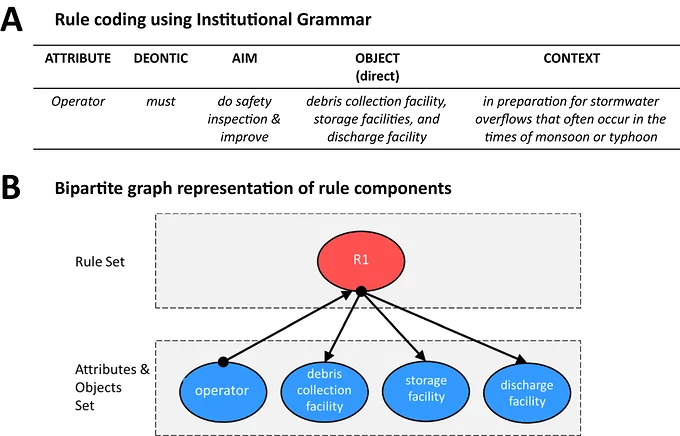
Illustrative institutional grammar example; the rule labeled R1 in this figure is a generic example and is unrelated to the Lake Mendocino rule numbering. (A) Decomposition of a rule statement into Institutional grammar components. (B) Bipartite graph representation of the coded rule components, with two node sets: rules and rule components (ATTRIBUTE and OBJECT only).

The value of this step lies not simply in making rules easier to read, but in making them comparable and analytically portable. Rules that appear in prose as routine administrative instructions are re‐expressed as relational elements linking actors, actions, targets, and operational conditions. For example, a rule requiring the US Army Corps of Engineers to establish and transmit a release schedule to the city of Ukiah can be coded to show both the direction of authority and the informational object being transmitted. Likewise, rules governing transitions between flood‐control and power‐generation modes can be coded to show how operational states depend jointly on hydrologic thresholds and institutional authority. In this way, rule coding reveals the control and coordination structure embedded in the text itself.

Several features of the Lake Mendocino rule corpus are especially important for the framework. Many rules are relational instructions connecting multiple actors and infrastructure objects rather than isolated commands. Many include conditional triggers tied to hydrologic or operational thresholds. Others specify communication requirements, including both message content and mode of transmission. These features matter because they shape how the rules are later represented in the STAMP control structure and how human error is parameterized in the Bayesian stage. The coding stage therefore produces more than a structured inventory of rules: it generates the formal representation of rule‐mediated relations required for subsequent STAMP and Bayesian analysis. The full Institutional Grammar coding for the rule corpus is provided in Table A1 in the Supporting Information.

### 2.4 STAMP‐Based Representation of Hazards, Control, and Failure

#### 2.4.1 Hazards and Safety Constraints

Once the rule corpus has been coded, the Lake Mendocino system is represented as a socio‐technical control architecture. Following STAMP, the analysis begins by identifying the system‐level hazards the control structure must prevent and the safety constraints required to keep operations within safe bounds. In the present study, hazards are defined with respect to unsafe states that could propagate across sectors and lead to one or more of three outcomes: failure of Coyote Valley Dam, impacts on downstream communities, or failure of Potter Valley Powerhouse Diversion Tunnel. These hazards anchor the remainder of the analysis: they define what the control structure is meant to prevent, guide the identification of ICAs, and later become the end nodes of the Bayesian model.

The corresponding safety constraints are derived jointly from the rule corpus and the physical system. Some are explicit, such as requirements to transmit release schedules, maintain monitoring, or operate under specified release modes. Others are implicit in safe dam operation, such as the need for reliable sensing, actuation, and feedback even when not stated directly in the rules. This matters because STAMP is not simply a theory of rule compliance, but of control: safety depends on whether the system maintains the constraints needed to keep operations within safe bounds. For example, a mode‐specific operating rule such as R14 (Table 1) does not merely describe an equipment setting; it also implies a hazard‐relevant control relation, the safety constraint that relation is intended to maintain, and the physical process through which unsafe conditions may emerge if the rule is not correctly followed (see Box B2 in the Supporting Information).

#### 2.4.2 SCS Construction

With hazards and safety constraints defined, the next step is to construct the SCS. The coded rules provide the mapping: ATTRIBUTEs identify controllers or responsible actors, OBJECTs identify controlled entities or targets, DEONTICs and AIMs specify the nature of rule‐governed action, and CONTEXTs identify the circumstances under which that action applies. These elements are placed within a hierarchical control structure that includes actors, infrastructure components, sensors, actuators, and feedback channels (Figure 4). In the Lake Mendocino case, the SCS includes actors such as the US Army Corps of Engineers, the city of Ukiah, and PG&E operators, together with dam gates, turbines, intake structures, release channels, monitoring points, and downstream receptors. Its purpose is not to catalogue all system components, but to represent the interfaces through which governing rules are translated into action and monitored through feedback.

**FIGURE 4:**
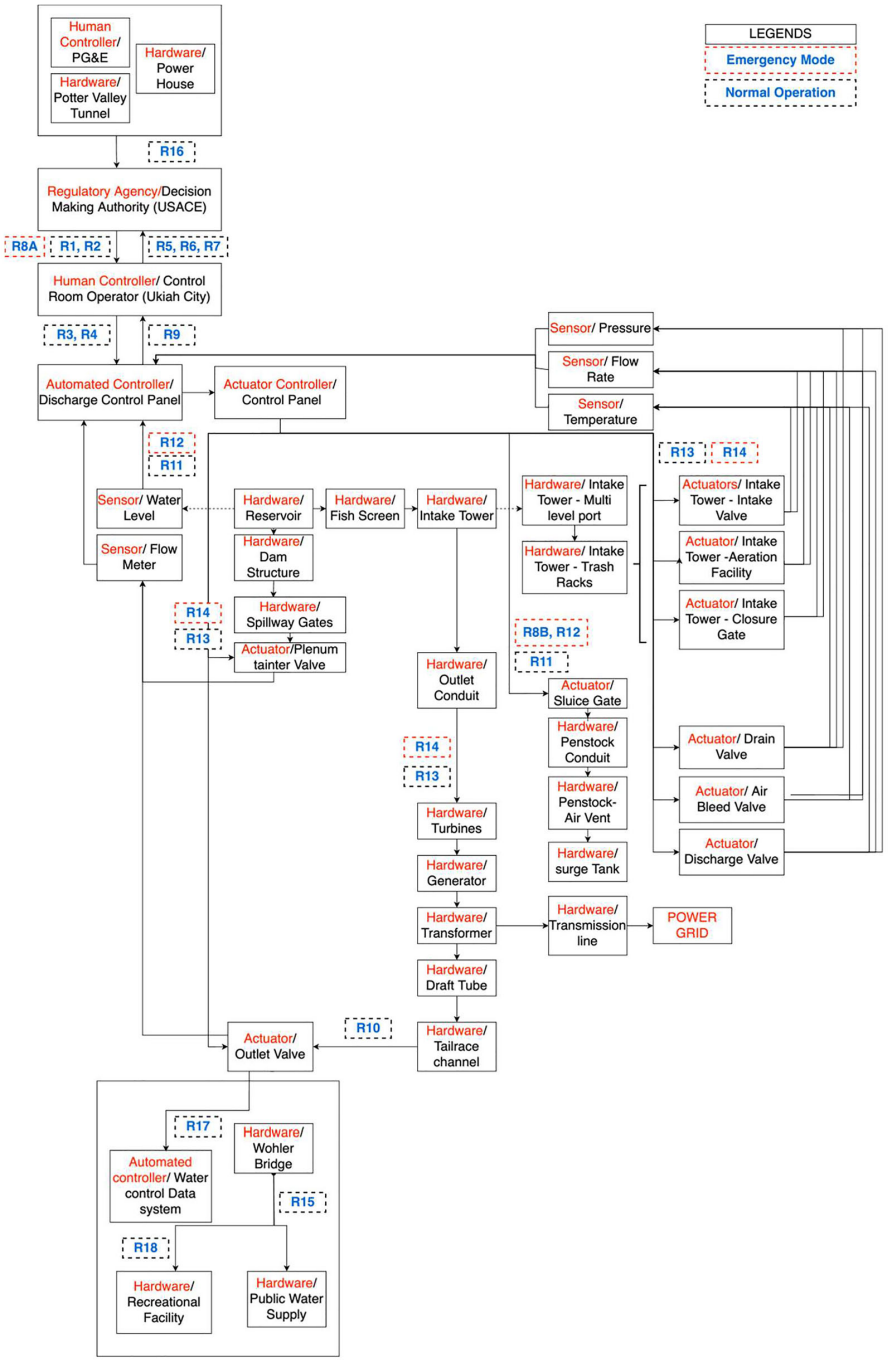
STAMP‐based hierarchical safety control structure (SCS) illustrating operational interdependencies and hazard propagation pathways in the Lake Mendocino water resource system. The diagram captures control loops and decision‐making authorities (USACE, Ukiah City operator), highlighting how emergency modes (red dashed boxes) and normal operations (blue dashed boxes) impact infrastructure, sensors, actuators, and downstream community functions.

A key modeling issue is that rules define protocols of control and coordination but do not explicitly enumerate every sensing and actuation mechanism required to implement them. A rule may require maintaining a dissolved oxygen threshold or continuous monitoring, for example, without specifying the sensors, interfaces, or physical channels needed to verify compliance. The framework therefore supplements the rule‐derived structure with physically necessary but rule‐implicit components drawn from reservoir and hydropower domain knowledge (Table A2 in the Supporting Information). This produces a structurally complete SCS that includes the physical and informational interfaces through which governing rules operate.

As shown in Figure 4, the resulting SCS depicts how decision‐making entities such as the US Army Corps of Engineers, city of Ukiah operators, and PG&E actors interface with physical infrastructure through control and feedback loops. It also differentiates between normal and emergency operating modes, making it possible to analyze how changes in operating mode alter the pattern of interdependencies and risk. A further contribution of the framework is that each coded rule is annotated directly onto the SCS interface it governs, making it possible to identify where rule coverage is absent or ambiguous and where control actions remain institutionally under‐specified (dashed rectangles in Figure 4).

#### 2.4.3 Identification of ICAs

Given the structure of human and non‐human controlling components in the system, STAMP identifies ICAs using the standard STAMP categories. At each relevant control interface, the analyst asks whether (1) a required action is not executed, (2) an unsafe action is provided, (3) a correct action is executed at the wrong time or in the wrong order, or (4) an action is applied too long or terminated too early (Leveson 2011). Applied to a rule corpus, this procedure becomes a structured method for converting governing rules into rule‐deviation scenarios with explicit safety implications.

Building on STAMP, our framework focuses on errors or mistakes in rule‐governed actions rather than attempting to enumerate every possible mechanical malfunction, software breakdown, or sensor defect in the Lake Mendocino system. The aim is to identify how deviations in rule‐governed actions (e.g., missed communications, mistimed releases, absent authorizations, unsafe directives, or other lapses and violations in carrying out prescribed protocols) may compromise safety constraints and propagate through the broader system. This scoping keeps the analysis centered on logical interdependencies and their hybrid combinations.

Each rule is interrogated through the four ICA categories, creating a systematic rule‐by‐rule inventory of possible control failures (see Box B3 in the Supporting Information for additional examples). For example, R14 requires reservoir operators to establish the flood‐control configuration by fully opening the plenum tainter valve, keeping the turbines and bypass valves fully closed, and opening the control gates as necessary. An ICA occurs if a required action is omitted, such as failing to fully open the plenum tainter valve, or if an unsafe action is provided, such as leaving the turbines or bypass valves open during flood‐control mode. Instead of relying on intuition to identify a few obvious failures, the method evaluates every rule against the same control taxonomy. The output is a set of rule‐level failure statements, each linked to a specific control interface and ICA type. The resulting rule‐deviation statements and SPAR‐H task classifications are summarized in Table A3 in the Supporting Information.

#### 2.4.4 Causal Factors and Hybrid Interdependency Motifs

The ICA inventory is then extended into an analysis of causal factors. For each rule‐based ICA, the framework traces the organizational, technical, or communication‐related conditions under which the deviation might occur and the pathways through which it could propagate. This step moves the analysis beyond identifying rule‐deviation types to asking why a rule‐based failure might occur and how it could matter systemically.

In the Lake Mendocino case, this analysis shows that the failures of interest rarely remain purely logical in their consequences. A communication failure may originate in human error in rule‐governed action, pass through an informational interface, and then produce an operational consequence in a physical component. Likewise, a delay in issuing or acting on a release schedule may begin as a rule deviation but become critical through its interaction with thresholds, gates, channels, or downstream infrastructure conditions.

To make these recurring cross‐domain patterns analytically tractable, the framework extracts hybrid interdependency motifs from the STAMP control structure. A network motif is defined here as a recurring network building block (Takes et al. 2018) in which a rule‐level failure at one control interface triggers effects that cross one or more other interdependency types (Yu et al. 2024). For example, a rule requiring communication of a release schedule may generate a motif in which a logical failure couples with an informational failure and eventually produces a physical consequence downstream. These motifs then provide the templates for specifying the causal edges in the BN. Figure 5 summarizes the common hybrid motif pattern identified from the Lake Mendocino control structure.

**FIGURE 5:**
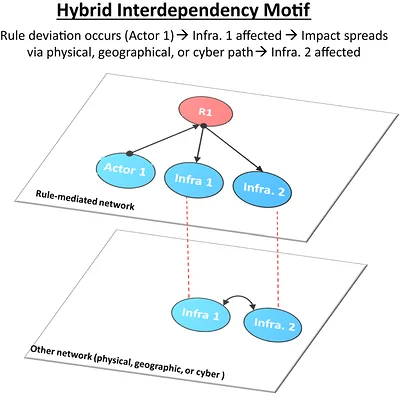
Hybrid interdependency motifs emerging from qualitative STAMP analysis, a rule error triggers an infrastructure impact coupled with geographical, cyber, or physical propagation, ultimately affecting downstream infrastructure.

### 2.5 Bayesian Translation and Quantitative Model Construction

#### 2.5.1 Network Scope, End Nodes, and Edge Structure

The Bayesian stage translates the qualitative outputs of the integrated institutional–STAMP analysis into a probabilistic model of systemic risk (Meng et al. 2022; Ozan Ceylan et al. 2025). The network is constructed to evaluate how rule deviations and related control failures propagate toward specific system‐level hazards. In the present case, the three end nodes correspond to the hazards identified in the STAMP stage: failure of Coyote Valley Dam, downstream community impacts, and failure of Potter Valley Diversion Tunnel. These end nodes define the outcomes the model is intended to explain and anchor the scope of the network.

The network contains four broad types of nodes: actor nodes, rule‐deviation nodes, infrastructure or object nodes, and end nodes. Actor nodes represent the agencies or operational entities whose decisions initiate control actions or cross‐infrastructure coordination. Rule‐deviation nodes represent the ICA‐based failures identified in the STAMP stage. Infrastructure or object nodes represent the physical or informational entities directly affected by those failures. End nodes represent the three system‐level hazards.

Each rule‐based ICA is translated into a rule‐deviation node and treated as a risk‐influencing factor in the BN. The model is therefore built not from generic failure factors, but from rule‐deviation scenarios grounded in the institutional arrangements and control structure (see Table A3 in the Supporting Information). The edge structure is then derived from the hybrid interdependency motifs extracted from the STAMP control structure. This keeps the Bayesian model faithful to the propagation pathways identified in the previous steps. For example, when a rule deviation affects a communication channel that then influences a physical object, the corresponding BN edges follow that same motif. In this way, the network formalizes the conditional structure of rule‐mediated and hybrid disruption pathways already identified qualitatively.

#### 2.5.2 Parameterization of Rule, Actor, and Infrastructure Nodes

Once the BN structure is defined, the model must be parameterized. Because the network represents a socio‐technical system, different parameterization strategies are used for different node types. Rule‐deviation nodes are parameterized using human reliability estimates, actor nodes are assigned aggregate probabilities based on the rules under their authority, and infrastructure object nodes are assigned failure probabilities using component reliability data. This combination allows the network to represent human and physical contributions to systemic risk within a single probabilistic structure.

Rule‐deviation nodes (based on R1–R18, with R8 represented separately as R8A and R8B, and ICA‐based failures) are parameterized using the Standardized Plant Analysis Risk–Human Reliability Analysis (SPAR‐H) method (Gertman et al. 2005). SPAR‐H, originally developed by the US Nuclear Regulatory Commission, provides a structured way to estimate human error probabilities (HEPs) for tasks under varying operational conditions and performance influences. Among available human reliability analysis approaches, SPAR‐H was selected because its structure fits the present application particularly well. It distinguishes diagnosis and action tasks, adjusts nominal HEPs using a limited set of explicit performance shaping factors, and produces quantitative HEPs that can be used as probabilistic inputs to the BN. Other established approaches, such as Technique for Human Error Rate Prediction (THERP; Swain 1983), Human Error Assessment and Reduction Technique (HEART; Williams 2015), or Cognitive Reliability and Error Analysis Method (CREAM; Hollnagel 1998), could also be applied, but SPAR‐H was considered a better match to the task representation, available information, and Bayesian parameterization used here. In the present model, the rule deviations are, in most cases, failures of diagnosis, action, or both. Diagnosis tasks involve situation assessment, information synthesis, and decision‐making under uncertainty; action tasks involve direct procedural or physical execution; and joint tasks involve both.

For the Lake Mendocino case, each rule‐governed action is first classified into one of these three SPAR‐H task categories. Some rule‐governed actions are primarily diagnostic, some are primarily action‐oriented, and others combine both. Rules such as R2 through R3, for example, require both interpretation of directives and procedural response and are therefore treated as joint tasks. Each rule $R_{i}$ is then evaluated using SPAR‐H's eight Performance Shaping Factors (PSF*s*): Available Time, Stressors, Complexity, Experience/Training, Procedures, Ergonomics/Human–Machine Interface, Fitness for Duty, and Work Process Quality (Table A4 in the Supporting Information). The product of individual factors $PSF_{j}$ becomes a composite multiplier (Equation 1) for rule $R_{i}$:

(1) $${\mathrm{PSF}}_{\mathrm{composite}}^{R_{i}}=\prod_{j=1}^{8}\mathrm{PS}F_{j}\;$$

In SPAR‐H, diagnosis tasks are assigned a nominal human error probability (NHEP) of $1.0\;\times \;10^{-2}$,​ whereas action tasks are assigned a NHEP of $1.0\;\times \;10^{-3}$​. The adjusted HEP for rule $R_{i}$ of task type t (${\mathrm{HEP}}_{t}^{R_{i}}$) is then calculated as

(2) $${\mathrm{HEP}}_{t}^{R_{i}}=\frac{\mathrm{NHEP}\times {\mathrm{PSF}}_{\mathrm{composite}}^{R_{i}}}{\mathrm{NHEP}\left({\mathrm{PSF}}_{\mathrm{composite}}^{R_{i}}-1\right)+1}\;$$

where task type t can be either diagnosis or action. Equation (2) prevents unrealistic probabilities above 1.0 when several adverse PSFs are present. For joint diagnosis–action tasks, component error probabilities were estimated separately using SPAR‐H and combined multiplicatively:

(3) $${\mathrm{HEP}}_{\mathrm{joint}\;\left(\mathrm{AND}\right)}^{R_{i}}=\left({\mathrm{HEP}}_{\mathrm{action}}^{R_{i}}\times {\mathrm{HEP}}_{\mathrm{diagnosis}}^{R_{i}}\right)$$

This combination (Equation 3) assumes independence and represents the joint occurrence of diagnosis and action errors. Failures involving only one component are not included in this local error estimate.

PSF assignments reflect the contextual demands of each rule‐governed action and were developed through a structured, document‐grounded expert‐judgment process. Several members of the author team had prior research experience with Coyote Valley Dam operations and its Water Control Manual, and the author team assigned the PSF levels using a common decision protocol developed through consensus to map operational features of each rule to the corresponding SPAR‐H levels. For example, emergency rule‐governed actions such as R8B are characterized by high stress, limited time, and elevated complexity, whereas monitoring actions such as R9 are performed under more favorable conditions and therefore yield lower error probabilities. The detailed decision rules are provided in Appendix A4 in the Supporting Information, and uncertainty in these assignments is evaluated through the Monte Carlo sensitivity analysis in Appendix D in the Supporting Information. The resulting HEPs provide the baseline rule‐deviation probabilities used to parameterize the BN:

(4) $$P_{\mathrm{deviation}}\left(R_{i}\right)={\mathrm{HEP}}_{t}^{R_{i}}\;$$

For example, R1 is treated as a diagnosis task. Its baseline PSF multipliers are Available Time = 1, Stressors = 1, Complexity = 2, Experience/Training = 1, Procedures = 1, Ergonomics/HMI = 1, Fitness for Duty = 1, and Work Processes = 0.8, giving a composite multiplier of 1.6. Applying Equation (2) to the nominal diagnosis HEP of 0.01 yields an HEP of 0.0159, rounded to 0.016. Following Equation (4), this value is used as the baseline HEP for parameterizing the R1 rule‐deviation node.

Actor nodes represent the organizations whose decisions initiate subsequent rule‐governed actions. Because each actor governs a distinct subset of rules, its prior probability of error is estimated as the mean of HEPs across the rules for which that actor is responsible:

(5) $$P_{\mathrm{error}}\left(A_{k}\right)=\frac{1}{n_{k}}\;\sum_{i=1}^{n_{k}}{\mathrm{HEP}}_{t}^{R_{i}}$$

where $A_{k}$​ is actor k, $n_{k}$ is the number of rules governed by that actor, and ${\mathrm{HEP}}_{t}^{R_{i}}$​ is the baseline HEP assigned to a rule deviations under that actor's responsibility. This aggregation (Equation 5) is intentionally simple and serves as an operational approximation of actor reliability within the network.

Infrastructure object nodes are parameterized using literature‐based component failure rates. Following prior studies, component failures are assumed to follow an exponential distribution, so the probability of failure of an infrastructure node $Z_{m}$ over time t is given by Equation 6:

(6) $$P_{\mathrm{failure}}\left(Z_{m}\right)=1-e^{-\lambda t},$$

where λ is the component‐specific failure rate (Table A5 in the Supporting Information). Because the component failure rates are reported on an hourly basis, we use *t* = 1 h as a common reference exposure interval for converting these rates into failure probabilities. This interval is also consistent with the short operational timescale of reservoir monitoring and control at Coyote Valley Dam. Water‐control procedures use regularly timed gage and flow observations, allow more frequent computations during flood operations when needed, and constrain changes in flood‐control releases on an hourly basis; several individual steps involved in changing operating conditions also occur on sub‐hourly timescales. The 1‐h interval therefore provides a reasonable reference window for coupling infrastructure conditions with rule‐governed human actions in the static Bayesian model, but it is not intended to represent the exact duration of every operational task or a complete operating‐mode transition. Using a common interval places the hardware probabilities on a consistent time basis for integration with the task‐level HEPs.

Together, these parameterization steps allow the network to represent how human error, organizational coordination, and infrastructure reliability jointly shape systemic risk across the Lake Mendocino system (see Boxes B4 and B5 in the Supporting Information for examples on SPAR‐H application and BN parameterization, respectively).

#### 2.5.3 Inference Setup

Once parameterized, the network supports two complementary forms of analysis. First, probability propagation from actor, rule‐deviation, and infrastructure nodes to the end nodes yields baseline estimates of system‐level risk under the modeled operating conditions. Second, backward reasoning conditions the network on an end node and propagates evidence through preceding nodes in the causal chain, allowing the analyst to identify which rule deviations, actors, and infrastructure conditions are most influential once a given failure is assumed to have occurred. Formally, Bayesian inference updates the probability of an event C given observed evidence E as:

(7) $$P\;\left(C|E\right)=\frac{P\left(E|C\right)\cdot P\left(C\right)}{P\left(E\right)}\;,$$

In Equation (7), P(C) is the prior probability of C, P(E∣C) is the likelihood of observing evidence E given C, and P(C∣E) is the posterior probability of C after incorporating the evidence.

This two‐part probabilistic analysis is central to the present study. Baseline probability propagation estimates aggregate system vulnerability under the modeled rule structure, whereas backward inference addresses a key question posed in the introduction: whether rules that appear routine under normal conditions can nevertheless become highly consequential when failure occurs. To compare posterior shifts systematically across the network, the study computes the ratio of variation (RoV) for each node:

(8) $$\mathrm{RoV}\;\left(X_{i}\right)=\frac{P\left(X_{i}|E\right)-P\left(X_{i}\right)}{P\left(X_{i}\right)}\;,$$

where $P(X_{i})$ is the baseline reference probability of node $X_{i}$ before evidence is introduced, and $P(X_{i}|E)$ is the posterior probability after conditioning on end‐state failure evidence E. For the rule‐deviation nodes analyzed here, the baseline reference probability is the SPAR‐H HEP used to parameterize the rule. Larger RoV values indicate nodes whose failure probabilities increase sharply under the conditioned scenario, marking them as stronger contributors to systemic failure. RoV values near zero indicate relatively weak links to the end event. Continuing the R1 example, its SPAR‐H HEP of 0.016 serves as the baseline reference probability for the R1 rule‐deviation node. After conditioning the network on Coyote Valley Dam failure, the posterior probability increases to 0.2264, which corresponds to an RoV of 13.15 using Equation (8). The Bayesian stage therefore serves not only as a quantification device, but also as the mechanism through which the framework distinguishes routine rule deviations from rule and control relations that are structurally important for systemic failure (see Appendix C in the Supporting Information for validation of the current Bayesian model).

Taken together, the steps in Section 2 produce an integrated representation of the Lake Mendocino system that links rule coding, STAMP‐based control analysis, and Bayesian inference. This representation enables the framework to move from the conceptual treatment of logical interdependencies to their systematic and quantitative assessment as sources of systemic risk.

## 3 Results and Analysis

### 3.1 Baseline Failure Probability

Using the quantified BN structure (Figure 6), we compute the overall probability of infrastructure failure, representing the baseline likelihood of failure under the modeled operating conditions and parameter assumptions before conditioning on any specific observations. This analysis yields a 7% baseline probability of Coyote Valley Dam failure and a 3% baseline probability of downstream community impacts. These values are model‐based estimates and are most useful for comparing relative levels of systemic risk across modeled conditions and pathways rather than as precisely calibrated estimates of real‐world failure frequency. While the Potter Valley Powerhouse Tunnel node shows negligible direct failure probability, its interactions through water release tunnel gates (captured in Rule R16) contribute indirectly to Coyote Dam vulnerabilities. Taken together, the results indicate that logical interdependencies and their hybrids can make non‐trivial contributions to modeled systemic risk even when the pathways through which they operate are less visible under routine conditions.

**FIGURE 6:**
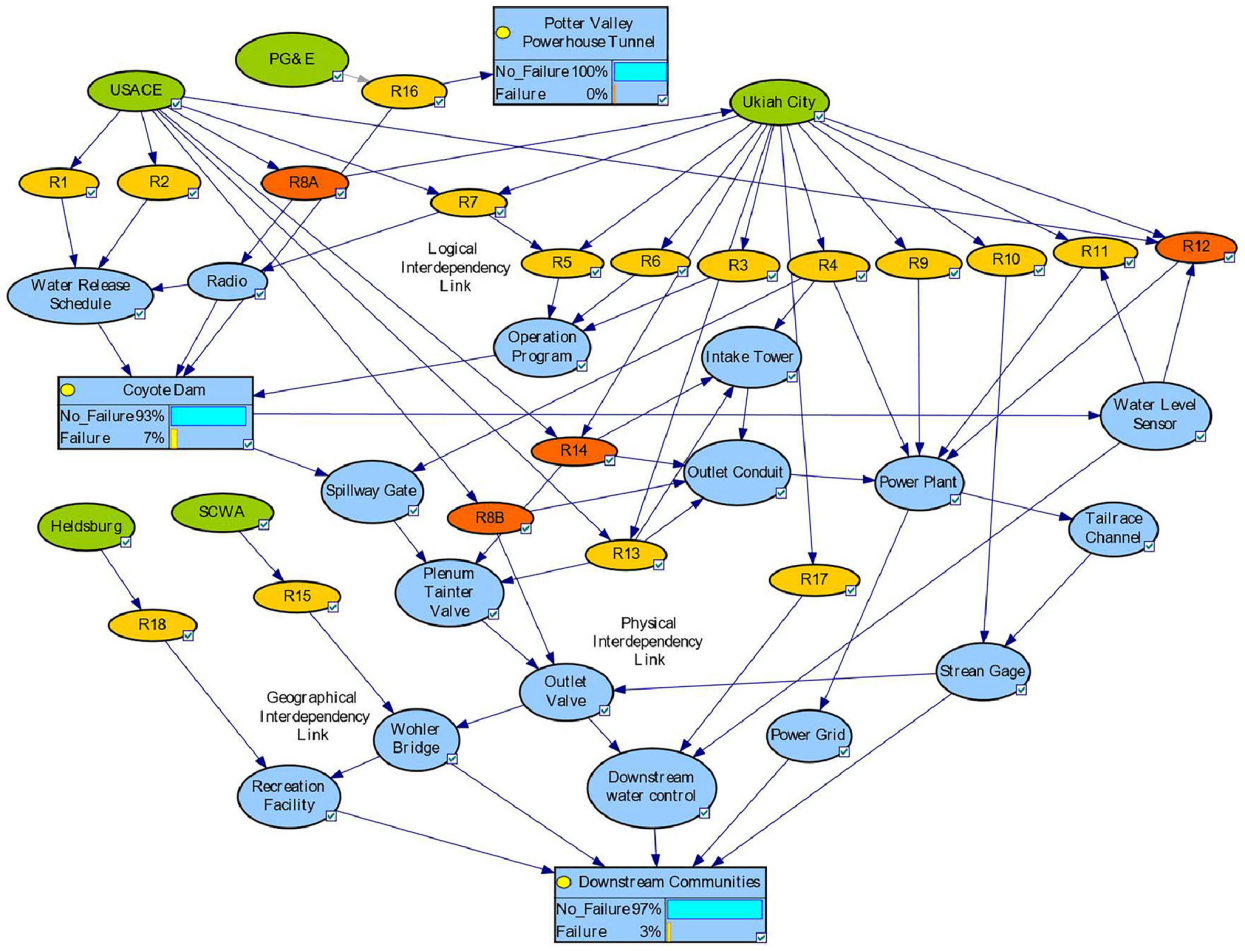
Bayesian network representation of the Lake Mendocino system used for quantitative risk assessment. Green nodes represent actors (e.g., USACE, Ukiah City), blue nodes represent infrastructure components (e.g., Coyote Dam, radio, stream gauge), yellow nodes denote operational rules under normal conditions (e.g., R1–R6), and red nodes denote emergency or contingency rules (e.g., R8A, R8B, R12, R14).

### 3.2 Baseline HEPs

The end‐node probabilities reported above indicate aggregate system risk, but they do not show which rule‐governed actions are most prone to failure. We therefore examine the baseline HEPs assigned to each rule‐deviation node, which serve as the model's input probabilities for human error in carrying out rule‐governed actions. To distinguish routine execution error from more substantial human‐reliability concerns, we use a significance threshold of $1\times 10^{-3}$. In safety‐critical settings, probabilities above 1 in 1000 are often treated as meaningful indicators that task demands, environmental stress, or coordination complexity exceed routine levels of manageability (Gertman et al. 2005).

As shown in Figure 7, the HEP distribution is highly uneven. On the logarithmic scale, six rules exceed the threshold of $1\times 10^{-3}$, and these higher values are concentrated primarily in emergency‐related rules. The highest baseline HEP is associated with R8A (Emergency Advisory Protocol), with an HEP of $2.4\times 10^{-1}$, indicating that this rule‐governed action has nearly a one‐in‐four chance of human error under emergency conditions. This value reflects the severe coordination burden, time pressure, and stress associated with emergency advisory communication across agencies. Two other emergency rules, R8B (Physical Intervention in Emergency) and R12 (Emergency Water Level Communication), also show elevated HEP values of $3.1\times 10^{-2}$ and $1.1\times 10^{-2}$, respectively, indicating that high‐pressure handoffs, rapid situational assessment, and time‐sensitive communication are especially vulnerable points in the rule system.

**FIGURE 7:**
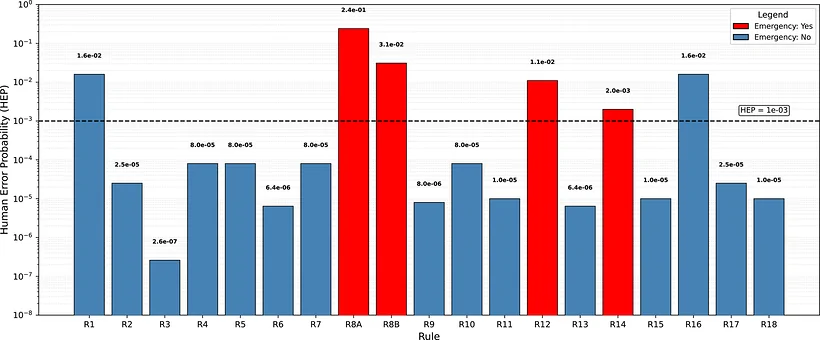
Human error probabilities (HEPs) for the 19 modeled rule‐deviation nodes corresponding to R1–R18 (with R8 split into R8A and R8B) on a logarithmic scale. Emergency‐related rules are highlighted separately from normal‐operation rules to show how baseline error propensity clusters within a limited subset of coordination‐ and stress‐intensive tasks.

Importantly, elevated HEP is not confined to overtly emergency‐related rule‐governed actions. R1 and R16 each exceed the threshold at $1.6\times 10^{-2}$. In both cases, the value appears to reflect diagnostic and coordination complexity: the need to interpret governing manuals, align decisions across dam operators, and translate institutional directives into operational action. R14 also crosses the threshold at $2.0\times 10^{-3}$, suggesting that backup protocols can become error‐prone when operators must respond quickly to primary‐system failure. Overall, baseline human error risk is not uniformly distributed across the control structure; it clusters around a limited subset of rules characterized by emergency pressure, communication complexity, or difficult cross‐organizational coordination.

### 3.3 Critical Rules Based on Backward Inference

Knowing which rule‐governed actions are most likely to involve human error is only half of the story. The other half is identifying which of those errors is actually decisive for systemic risk. To distinguish between these possibilities, we use backward Bayesian inference and the RoV metric. RoV measures how much a node's probability changes when the model is conditioned on Coyote Valley Dam failure. Table A6 in the Supporting Information reports baseline reference HEPs and posterior probabilities for human error in each rule‐governed action, and Figure 8 visualizes the corresponding RoV values.

**FIGURE 8:**
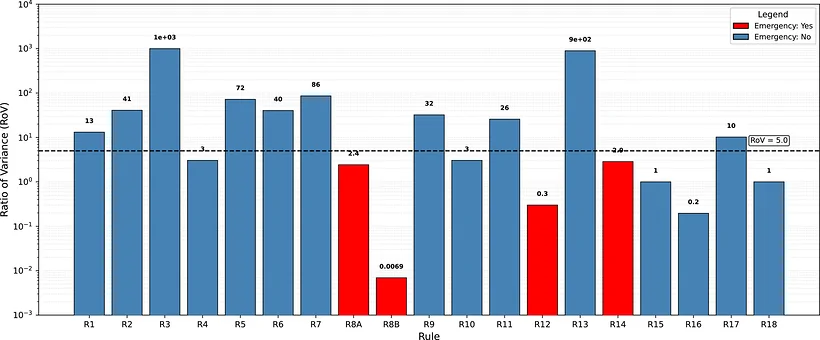
Sensitivity of rule‐violation nodes to the Coyote Valley Dam failure condition, expressed as ratio of variation (RoV). Higher values indicate rules whose posterior probabilities increase sharply once the system is conditioned on dam failure.

To isolate the most influential rule‐governed actions, we adopt an RoV threshold of 5.0. Under this formulation, the posterior probability of human error is at least six times its baseline reference HEP once the model is conditioned on Coyote Valley Dam failure. Although RoV is not a Bayes factor, this cutoff is consistent with Bayesian interpretive conventions in which posterior‐to‐prior shifts of three to ten times are treated as substantial evidence of changed probabilistic importance (Jeffreys 1998; Kass and Raftery 1995). Under this criterion, 10 rule‐governed actions emerge as important contributors to the modeled failure pathway.

At the point of physical execution, R13 (the rule governing control gate operation) emerges as the one of the most consequential rule‐governed action, with RoV = 895.24. Although human error in carrying out this action is not especially likely under baseline conditions, it becomes overwhelmingly salient once the model is conditioned on Coyote Valley Dam failure. Because gate operation sits so close to the dam's physical dynamics, even small execution errors can propagate with little opportunity for correction.

Rest of the rules reveal a second pattern centered on authority, information exchange, and translation into local operation. R1 acts as a structural bottleneck by governing how release authority is established and translated into decisions. That sensitivity then propagates through R2 and R7, which govern the transmission of release intent and schedule information. Together, these rules define a logical interdependency pathway through which breakdowns in authority and information exchange propagate toward physical consequence. R3 captures the possibility that a correctly issued schedule or directive still fails to produce safe local operation. Finally, the remaining critical rules (R5, R6, R9, R11, and R17) reinforce this pathway by governing the continuous monitoring, strict operational mode boundaries, and communication feedback loops necessary to sustain that schedule integrity.

The contrast between R8A (RoV = 2.4) and R8B (RoV = 0.00694) is especially revealing. Although both are emergency rules, the advisory protocol (R8A) has much greater importance in the modeled failure pathway than the rule granting physical intervention authority (R8B). This suggests that system safety in emergency mode is more sensitive to coordination and advisory communication than to the formal existence of emergency override authority alone.

### 3.4 Risk Quadrant Analysis

The HEP and RoV analyses capture two different dimensions of systemic risk. HEP identifies which rule‐governed actions are most prone to human error under baseline operating conditions. RoV identifies which of those errors become most consequential in the modeled failure pathway once the model is conditioned on Coyote Valley Dam failure. To integrate these dimensions, Figure 9 plots HEP against RoV for all eighteen rules in a four‐quadrant risk map. As shown below, the quadrant analysis reveals *hidden logical risk*: rules and control relations that appear routine in normal operation yet become disproportionately consequential in the modeled failure pathway.

**FIGURE 9:**
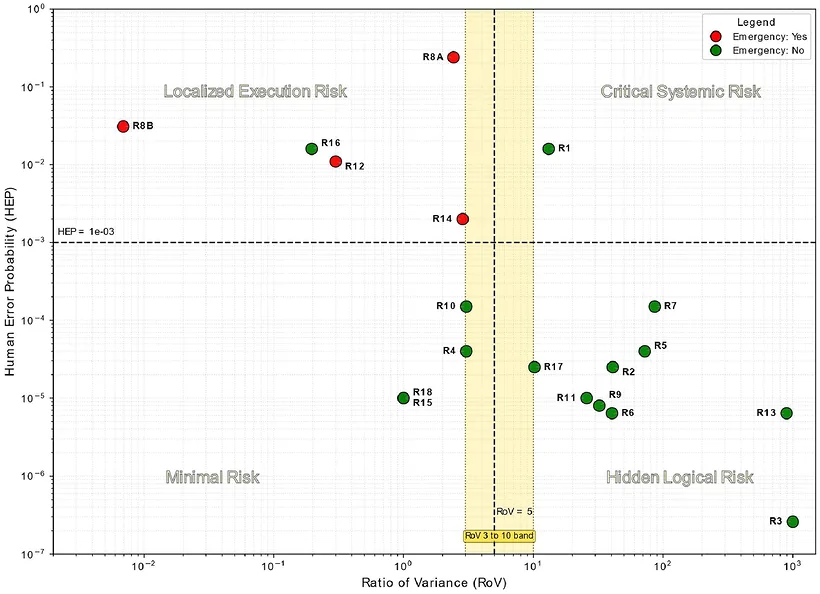
Four‐quadrant map of human error probability (HEP) versus ratio of variation (RoV) for rule‐violation nodes. The vertical axis shows baseline error propensity derived from SPAR‐H; the horizontal axis shows structural sensitivity under the Coyote Valley Dam failure condition. The vertical and horizontal dashed lines represent the significant thresholds for RoV and HEP, respectively. The four quadrants distinguish critical systemic risk, localized risk, minimal risk, and hidden logical risk.

*The “critical systemic risk” quadrant (high HEP, high RoV)*: This quadrant contains rule‐governed actions that are both prone to human error and strongly involved in the modeled failure pathway. In the present analysis, R1 occupies this quadrant, making it the clearest priority for ensuring reliable execution and reducing error.

*The “localized execution risk” quadrant (high HEP, low RoV)*: Rule‐governed actions in this quadrant are relatively error‐prone, but their posterior probabilities increase less sharply when the model is conditioned on dam failure. R8A, R8B, R12, R14, and R16 fit this profile. They therefore indicate substantial execution demands, but weaker posterior amplification in the modeled failure pathway.

*The “minimal risk” quadrant (low HEP, low RoV)*: Four rule‐governed actions occupy this quadrant. They show neither elevated baseline error propensity nor strong posterior sensitivity once the model is conditioned on dam failure. These actions make up much of the proceduralized operating background of the system and, under the present model, do not emerge as priority targets for intervention.

*The “hidden logical risk” quadrant (low HEP, high RoV)*: This quadrant is the most analytically distinctive result of the framework. R2, R3, R5, R6, R7, R9, R11, R13, and R17 all have relatively low baseline error probabilities under normal conditions, yet their RoV values increase sharply once the model is conditioned on dam failure. These are precisely the kinds of rule‐mediated vulnerabilities that conventional prospective risk assessment is likely to miss: routine actions that are not especially error‐prone under normal conditions, yet become highly consequential once failure propagates through the system. This quadrant gives concrete meaning to one of the paper's central claims: some logical interdependencies remain hidden not because they are unimportant, but because they appear reliable until stress reveals their structural significance.

A Monte Carlo sensitivity analysis (Appendix D in the Supporting Information) evaluated the robustness of these findings over 1000 iterations. Available Time, Complexity, Procedures, and Work Processes were sampled from rule‐specific ranges around their baseline SPAR‐H levels using a triangular distribution centered on the baseline; stressors was derived from sampled Available Time and Complexity; and Experience/Training, Ergonomics/HMI, Fitness for Duty, and infrastructure failure rates were held fixed. In each iteration, HEPs and actor priors were recalculated, the HEP‐derived Bayesian parameters were updated, and forward and backward inference were rerun to reclassify each rule. The hidden logical risk rules remained in that quadrant in 70.2%–98.0% of iterations. Under triangular sampling, Coyote Valley Dam failure probability exceeded 1% in 97.2% of iterations and Downstream Communities failure probability exceeded 1% in 97.7%. These results indicate that the principal qualitative findings are robust to plausible variation in the human/rule parameterization, even though absolute failure probabilities remain sensitive to PSF assumptions.

## 4 Discussions

### 4.1 Logical Interdependencies as Rule‐Mediated Relations

A central theoretical implication of this study is that logical interdependencies are best understood not as a residual category of infrastructure connection, but as rule‐mediated relations that shape systemic risk. Rinaldi et al. (2001) introduced the category to describe dependencies arising through human decisions, but left it analytically broad. Yu et al. (2024) sharpened the concept by grounding it in institutional arrangements and rule‐mediated motifs. The present analysis pushes the argument further by showing that rule‐mediated logical interdependencies are not simply links among infrastructures; they are rule‐governed control and coordination relations through which system safety is maintained or lost.

STAMP provides the analytical structure for this argument. Once rules are embedded in a control structure, logical interdependencies no longer appear as vague, leftover connections among infrastructures. They become part of a governance‐bearing architecture of control, feedback, and constraint enforcement. From this perspective, failures propagate not only through material or informational dependencies, but also through delayed, omitted, mistimed, or poorly coordinated rule‐governed actions. Logical interdependencies thus become hazard‐relevant in a more precise sense: depending on how control is exercised, they can either keep the system within safe bounds or help push it toward unsafe states. Rules, in this formulation, function much like valves, channels, and gates: they organize flows, but the flows are those of authority, information, timing, and obligation. The framework makes that soft infrastructure visible by locating rules within a socio‐technical control structure rather than treating them as text alone.

The Lake Mendocino case illustrates this argument. The decisive pathway does not begin at visible physical failure, but earlier in the chain through which release authority is established, conveyed, interpreted, and translated into action. R1, R2, R7, and R3 are therefore not merely administrative rules sitting outside the system; they are part of the system's control logic. They matter not because they are dramatic, but because they occupy critical positions in pathways of authorization, communication, and execution. R13 matters for a different reason: it sits where coordinated control is translated into immediate hydraulic effect. The broader implication is that rules become consequential not simply because they differ by type, but because of where they sit in socio‐technical pathways through which failure spreads across infrastructures.

### 4.2 Hidden Logical Risk as a Resilience Blind Spot

A central finding of the analysis is that hidden logical risk constitutes a resilience blind spot. The results show that baseline error propensity and system‐level consequence do not align neatly: some rule‐governed actions are difficult to execute, while others appear reliable under normal conditions yet become highly consequential once the model is conditioned on failure. The system's most critical dependencies are therefore not always the ones most likely to fail in routine operation.

This point matters beyond the present case. Infrastructure resilience research often emphasizes exposure, redundancy, restoration, and visible cross‐sector dependencies, while SETS research highlights the coupling of social, ecological, and technical processes. The present findings add a more specific insight to both conversations: resilience can be undermined by less visible bottlenecks embedded in rule‐mediated coordination. In the Lake Mendocino case, R2, R3, and R7 show how routine information and coordination rules gain systemic importance as the system moves toward failure; continuous‐monitoring and boundary rules (R5, R6, R9, R11, and R17) reveal vulnerabilities in sustaining safe operating conditions; and R13 shows how a routine control rule becomes decisive when coordinated action is translated into physical release. These are easy to overlook because they do not appear as conventional infrastructure vulnerabilities until failure exposes them. From this perspective, hidden logical risk is not peripheral to resilience analysis; it is one way in which coupled socio‐technical and social‐ecological‐technical systems become fragile under stress.

By connecting specific rules to control failures and modeled consequences, the framework brings these governance dependencies into risk analysis and helps assess whether governance arrangements fit the infrastructure dependencies they are meant to manage (Yu et al. 2020). In this way, risk analysis can identify not only how disruption may propagate, but also where coordination is vulnerable and where changes to rules or their implementation may strengthen the collective capacity to respond and adapt under stress—capacities that are central to resilience. The framework does not measure those resilience gains directly, but it identifies where they may be most needed.

### 4.3 Rules as Design Variables—Risk Quadrant‐Informed Intervention Targets

A practical strength of the framework is that it turns rules into design variables for risk reduction. The distinction between HEP and RoV is useful because it separates rule‐governed actions that are difficult to carry out from control relations that are structurally important for system failure, and therefore points to different intervention targets. Rule‐governed actions with high HEP and high RoV, such as R1, indicate priorities for procedural redesign, decision aids, and stronger coordination support. Those with high HEP but low RoV, such as R8A, R8B, R12, R14, and R16, point instead to demanding actions that may benefit most from training, simplification, and operational support. By contrast, actions with low HEP but high RoV, such as R13 and the other pathways involving the rules in the hidden risk quadrant, identify structurally important control relations that may require redundancy, cross‐checking, monitoring, or redesign even if they do not appear problematic in routine performance measures.

The Lake Mendocino case illustrates how such findings could inform practical rule redesign. R7, for example, currently requires release schedules, operations programs, and revisions to be transmitted by telephone or radio. Because R7 appears in the hidden logical risk quadrant, an intervention could focus not simply on improving operator training but on redesigning the communication rule itself, for example by adding a digital or SCADA‐based transmission channel, automatic logging, explicit acknowledgment of receipt, and a redundant backup channel when confirmation is not received. R13 suggests a different type of intervention. Because it governs the configuration of gates, valves, and turbines through which coordinated decisions are translated into physical releases, the rule could be strengthened by requiring independent confirmation of equipment state, such as a second‐operator check or automated comparison between commanded and observed gate and valve positions. These examples are illustrative rather than prescriptive: the present analysis identifies where redesign may have the greatest value, while evaluating the effectiveness of a specific intervention would require re‐parameterizing or extending the model to represent the modified rule and control structure.

The broader practical implication is that agencies should not focus only on hard assets, but also audit the control architecture linking organizations to operations. Questions about who authorizes releases, how schedules are transmitted, how feedback is received, when emergency mode is activated, and how transfers of responsibility are verified are part of the risk structure itself. From this perspective, safer infrastructure systems will often require redesigning the control architecture, not only upgrading the hardware.

### 4.4 Scope, Limitations, and Transferability

The current study has several clear limitations. First, because of our focus on a formal rule corpus, our results do not capture effects of informal rules, tacit norms, discretionary improvisation, or workarounds that may also shape system behavior. Second, actor nodes are intentionally aggregated, with their prior error probabilities estimated as the mean HEPs of the rules under their authority. This keeps the Bayesian model tractable but couples actor‐ and rule‐level parameterization and compresses potentially important variation within organizations and roles. Future work could estimate actor‐level reliability independently using organizational or operational data. Third, the probabilistic model is static and event‐conditioned, making it well suited to diagnosing propagation structure and hidden bottlenecks but less suited to representing adaptation, learning, repeated interaction, or endogenous rule revision during unfolding events. Fourth, the case boundary is deliberately tractable rather than basin‐complete, so the model represents a critical control architecture rather than every interaction in the wider basin. Fifth, for rules classified as joint diagnosis–action tasks, the estimated HEP depends on how diagnosis and action errors are combined. Excluding single‐component failures may underestimate overall task‐error probabilities where either component can independently violate a rule, potentially affecting risk classifications, including hidden logical risk. The sensitivity analysis examines parameter variation within this formulation. Future work should assess alternative error combinations and dependence between components. A final limitation concerns the interpretation of the quantitative estimates. Some model inputs, particularly the SPAR‐H PSF assignments, involve analyst judgment and uncertainty. The resulting failure probabilities should therefore not be interpreted as precisely calibrated estimates of real‐world failure frequency. These estimates are most useful in a comparative and diagnostic sense: they allow us to examine how modeled risk differs across rule‐governed actions, pathways, and operating conditions, and to identify institutional‐control relationships that contribute disproportionately to systemic risk. The sensitivity analysis in Appendix D in the Supporting Information evaluates the extent to which these findings remain robust under reasonable variation in the PSF assignments.

These limitations also clarify where the framework is most transferable. Its transferability is primarily methodological rather than empirical. The analytical framework can be applied to other infrastructure systems in which safety depends on formal operating rules, distributed authority, recurring inter‐organizational coordination, and identifiable interfaces between institutional action and physical consequence. Reservoir systems are one example, but the same analytical logic may apply to emergency management, interconnected transportation systems, and other governed infrastructure systems. The empirical results, however, are case‐specific. The probability estimates, RoV values, rule rankings, and identified disruption pathways depend on the particular rule corpus, organizational structure, physical system, and parameterization of the Lake Mendocino case and should not be assumed to transfer directly to other systems or regions. The framework is therefore not intended as a fully calibrated predictive model of system failure. Its main value is diagnostic: making governance‐mediated failure pathways visible, comparing their relative importance, and identifying potentially correctable vulnerabilities before disruption occurs.

## 5 Conclusions

This paper shows that systemic risk in critical infrastructure cannot be understood only through material, spatial, or informational interdependencies. It must also be understood through the rule‐mediated relations by which infrastructures are controlled and coordinated. From this perspective, critical infrastructures fail not only when components break or physical dependencies transmit disruption, but also when authority is misaligned, communication delayed, timing mistuned, or control actions translated poorly across organizational and infrastructural boundaries.

Methodologically, the framework links institutional analysis, STAMP‐based control analysis, and Bayesian inference to identify vulnerabilities that conventional approaches are likely to miss: rules and control relations that appear routine in normal operation yet become decisive under failure conditions. In the Lake Mendocino case, this makes it possible to distinguish between rules that are demanding to execute, rules that are structurally decisive for system safety, and hidden logical risks that would remain obscured in analyses focused only on physical interdependence or routine error likelihood. The contribution is therefore not simply to represent governance more fully, but to diagnose how rule‐mediated relations become pathways of systemic risk and where changes to rules or control relations could reduce vulnerability.

The framework is best suited to settings in which formal operating rules, distributed authority, and identifiable interfaces between institutional action and physical consequence play a central role. It is less able to capture informal practices, adaptive learning, and endogenous rule revision during unfolding events. Future work should extend the approach by incorporating informal and tacit institutions, representing changing control relations over time, and testing whether similar forms of hidden logical risk arise in other infrastructure domains.

## Conflicts of Interest

The authors declare no conflicts of interest.

## Supporting information




**Supporting Information**: risa70387‐supp‐0001‐SuppMat.docx
