# Supplementary material for: Modeling Logical Interdependencies: Quantifying Systemic Risk From Rule‐Governed Actions
Source: Risk Anal. 2026 Oct 14;46(11):e70387. doi: 10.1111/risa.70387 (PMC13669526; doi:10.1111/risa.70387)
Supplement: Supplementary file 1 [file RISA-46-0-s001.docx]

*Supplementary material for*

**Modeling Logical Interdependencies: Quantifying Systemic Risk from Rule-Governed Actions**

**Appendix A**

**Table A1**: Structured parsing of institutional rules governing Lake Mendocino operations using the ABDI grammar. Each rule is decomposed into four main syntactic elements: Attribute, Deontic, aIm, oBject, enabling formal integration into the HyRIM framework for risk modeling and control structure analysis. We use square bracket to indicate expressions that are not directly found in the rule statements but are implied based on the context. We do not code context and this means any physical entities mentioned in the context are not coded.

| **Rule No.** | **Rule Statements** | **Attribute** | **Deontic** | **aIm** | **oBject** |
| --- | --- | --- | --- | --- | --- |
| R1 | All water releases from Coyote Dam will be determined, controlled, and directed by the Carps. | [USACE] | will | determine, control and direct | Water Releases |
| R2 | The Corps will establish, and transmit to the City, dam flow release schedules (except during flood control operation) at the Coyote Dam for a weekly, daily, or other period as needed, in units of cubic feet per second. | [USACE] | will | establish and transmit | [Water Releases] |
| R3 | The City, after receiving the flow release schedules, shall provide an operations program for that period. | [Ukiah City] | shall | provide | Operations Program |
| R4 | The operation program will include the flow distribution through all discharge valves and the power plant necessary to comply with the Corps release schedules. | [Ukiah City] | will | include | Flow distribution through all discharge valves and the power plant |
| R5 | The Corps will be notified immediately of any revisions to this program. | [Ukiah City] | will | [notify] | Revisions to [Operation Program] |
| R6 | In no case will such revisions affect the Corps release schedules. | [Ukiah City] | [must not] | [make] | Revisions to [Operations Program that] affect the Corps release schedules |
| R7 | The dam release schedules; operations program and revisions to the program will be transmitted by telephone or radio. Radio communication will be between the city and the Corps located in Sacramento, California on the City's supplied equipment and frequency. | [Ukiah City & USACE] | will | Transmit, [use] | [information (including schedules, program, revisions)],  radio/telephone |
| R8A | (c) During emergency situations, as determined by the Corps, the Corps will advise the city of the situation | [USACE] | will | Advise, [use] | [Ukiah City], [radio/telephone] |
| R8B | and may take appropriate action, including shutting down the power units and assuming control of the appropriate outlet gates and valves. | [USACE] | may | take | Action on power units, outlet gates and valves. |
| R9 | Plant operation will be monitored 24 hours per day | [Ukiah City] | will | monitor | Plant Operation |
| R10 | (f) The city will be responsible for maintaining a minimum dissolved oxygen level of 7.0 mg/l at the existing U.S.G.S. stream gage immediately downstream of the proposed tailrace | [Ukiah City] | [must] | maintain | A minimum dissolved Oxygen Level of 7.0 mg/l |
| R11 | Power generation mode will be in operation when the required dam releases are between 0 and 4375 cfs, inclusive and the flood pool is at or below elevation 755 feet. | [Ukiah City] | will | operate | [Power Plant] in power generation mode |
| R12 | Flood control mode will be in operation when the required dam releases exceed 4375 cfs, the flood pool exceeds elevation 755 feet, or the conservation pool exceeds elevation 762 feet. | [Ukiah City & USACE] | will | operate | [Power Plant] in flood control mode |
| R13 | (h). When the dam and power plant facilities are operating in the power generation mode, the control gates (three service slide gates and three emergency slide gates in the intake tower) will be fully open, the plenum tainter valve will be fully closed, and the turbines and bypass valves will be opened as necessary to pass the required water release | [Ukiah City & USACE] | will | [open], [close], [open] | [Intake tower],  plenum tainter valve,  turbines and bypass valves [in power generation mode] |
| R14 | (i) When the dam and power plant facilities are operating in the flood control mode the plenum tainter valve will be fully open, the turbines and bypass valves will be fully closed, and the control gates will be opened as necessary to pass the required water release. | [Ukiah City & USACE] | will | [open], [close], [open] | Plenum tainter valve,  turbines and bypass valves, [intake tower] [in flood control mode] |
| R15 | SCWA diverts municipal water at its pumping stations in the vicinity of Wohler Bridge below Healdsburg. | SCWA | [will] | divert | Municipal Water |
| R16 | Pacific Gas and Electric Co. (PG&E) divert majority of the inflow water to the Lake Mendocino via the Potter Valley powerhouse diversion tunnel. | PG&E | [will] | Divert, [use] | Majority of the inflow water to the [Coyote Dam], Potter Valley power house diversion tunnel |
| R17 | Coyote Valley operators will provide routine and emergency instructions and support for Water Control Data Systems (WCDS) | [Ukiah City] | will | provide | Routine and emergency instructions and support for Water Control Data Systems (WCDS) |
| R18 | The city strongly supports the maintenance of maximum summer flows in the Russian River to protect water quality and the recreational values of the Russian River. | [Healdsburg] | [must] | supports | Maintenance of maximum summer flows in the Russian River |

**Table A2.** Basic components and processes in reservoir and hydropower systems

Basic components and processes

1. **Reservoir**: Stores water at a higher elevation to create potential energy for electricity generation
2. **Dam Structure**: Controls the flow of water and maintains the reservoir's stability
3. **Spillway Gates**: Discharge excess water during floods to prevent overflow
4. **Intake Tower**: The intake tower in a reservoir is a critical structure that controls the flow of water for hydroelectric power generation, water supply, or other uses
   a. Multilevel Ports
      i. Located at various depths to draw water from different levels of the reservoir.
      ii. Designed for temperature and water quality management (e.g., controlling dissolved oxygen levels).
   b. Trash Racks: Installed at each intake port to prevent debris and floating materials from entering the system.
   c. Fish Screen: Movable screens installed at intake ports to protect aquatic life.
   d. Intake valve: Regulate water flow through intake ports.
   e. Energy Dissipation Valves: Release water during emergencies to avoid damage due to high pressure.
   f. Closure Gate: Used for tunnel closure or flow control in emergency situations.
   g. Outlet Conduit: Channels water from the intake tower to downstream systems such as penstocks or pipelines.
5. **Penstock**: Directs water flow with controlled pressure to turbines for hydroelectric generation
   a. Surge Tank: Absorbs pressure surges (water hammer) caused by sudden changes in turbine operation or valve adjustments.
   b. Sluice Gates: Positioned at the start of the penstock to regulate water flow entering the pipe.
   c. Air Vents: Prevent collapse by equalizing pressure when internal pressure drops too low
   d. Drain Valves: Installed along the penstock to release water during maintenance or emergency
   e. Air Bleed Valves: Prevent vacuum formation inside the penstock during sudden flow changes.
6. **Turbine:** Converts kinetic energy of moving water into mechanical energy
7. **Generator:** Converts mechanical energy from turbines into electrical energy using rotating magnets in a stationary magnetic field
8. **Transformer:** Steps up voltage generated by the power plant for efficient transmission over long distances
9. **Draft Tube:** Channels water exiting into the tailrace, reducing velocity and ensuring smooth discharge
10. **Tailrace channel:** Returns water to downstream rivers or reservoirs after passing through turbine

**Table A3.** SPAR-H categorization of Coyote Dam rule violations into diagnosis, action, or both. This table presents the first step of the SPAR-H (Standardized Plant Analysis Risk–Human Reliability Analysis) method by classifying each rule violation in Coyote Dam operations as a diagnosis**,** action**,** or both.

| **Rule No.** | **Description of rule violation** | **Categorize [SPAR-H]** |
| --- | --- | --- |
| R1 | All water releases from Coyote Dam may fail to be determined, controlled, and directed by the Corps. | Diagnosis |
| R2 | The Corps may fail to establish, and transmit to the City, dam flow release schedules (except during flood control operation) at the Coyote Dam for a weekly, daily, or other period as needed, in units of cubic feet per second. | Diagnosis + Action |
| R3 | The City, after receiving the flow release schedules, may fail to provide an operations program for that period. | Diagnosis + Action |
| R4 | The operation program may fail to include the flow distribution through all discharge valves and the power plant necessary to comply with the Corps release schedules. | Action |
| R5 | The Corps may fail to get notified immediately of any revisions to this program. | Action |
| R6 | Such revisions may affect the Corps release schedules. | Diagnosis + Action |
| R7 | The dam release schedules; operations program and revisions to the program may fail to be transmitted by telephone or radio. Radio communication will be between the city and the Corps located in Sacramento, California on the City’s supplied equipment and frequency. | Action |
| R8A | During emergency situations, as determined by the Corps, the Corps may fail to advise the city of the situation | Diagnosis |
| R8B | and may not take appropriate action, including shutting down the power units and assuming control of the appropriate outlet gates and valves. | Action |
| R9 | Plant operation may fail to be monitored 24 hours per day | Action |
| R10 | (f) The city may fail to maintaining a minimum dissolved oxygen level of 7.0 mg/l at the existing U.S.G.S. stream (stre gage immediately downstream of the proposed tailrace | Action |
| R11 | Power generation mode may fail to be in operation when the required dam releases are between 0 and 4375 cfs, inclusive and the flood pool is at or below elevation 755 feet. | Diagnosis + Action |
| R12 | Flood control mode may fail to be in operation when the required dam releases exceed 4375 cfs, the flood pool exceeds elevation 755 feet, or the conservation pool exceeds elevation 762 feet. | Diagnosis + Action |
| R13 | (h). When the dam and power plant facilities may fail to be operating in the power generation mode, the control gates (three service slide gates and three emergency slide gates in the intake tower) will be fully open, the plenum tainter valve will be fully closed, and the turbines and bypass valves will be opened as necessary to pass the required water release | Diagnosis + Action |
| R14 | (i) When the dam and power plant facilities may fail to be operating in the flood control mode the plenum tainter valve will be fully open, the turbines and bypass valves will be fully closed, and the control gates will be opened as necessary to pass the required water release. | Diagnosis + Action |
| R15 | SCWA may fail to divert municipal water at its pumping stations in the vicinity of Wohler Bridge below Healdsburg. | Diagnosis + Action |
| R16 | Pacific Gas and Electric Co. (PG&E) fail to divert majority of the inflow water to the Lake Mendocino via the Potter Valley powerhouse diversion tunnel. | Diagnosis |
| R17 | Coyote Valley operators may fail to provide routine and emergency instructions and support for Water Control Data Systems (WCDS) | Diagnosis + Action |
| R18 | The city fails to support the maintenance of maximum summer flows in the Russian River to protect water quality and the recreational values of the Russian River. | Diagnosis + Action |

**Table A4.** Baseline SPAR-H Performance Shaping Factor (PSF) assignments for each rule-governed action in Coyote Valley Dam operations. Each row reports the assigned PSF levels, task type (Diagnosis, Action, or Joint), and resulting composite multiplier. The basis for these assignments is described in the accompanying text following the table.

| **Rule No.** | **Emergency** | **Available Time** | **Stressors** | **Complexity** | **Experience** | **Procedures** | **Ergonomics** | **Fitness for Duty** | **Work Processes** | **Task Type** | **Composite Multiplier** |
| --- | --- | --- | --- | --- | --- | --- | --- | --- | --- | --- | --- |
| R1 | No | 1 | 1 | 2 | 1 | 1 | 1 | 1 | 0.8 | Diagnosis | 1.6 |
| R2 | No | 1 | 1 | 2 | 1 | 1 | 1 | 1 | 0.8 | Joint | 1.6 |
| R3 | No | 1 | 1 | 2 | 1 | 0.1 | 1 | 1 | 0.8 | Joint | 0.16 |
| R4 | No | 1 | 1 | 1 | 1 | 0.1 | 1 | 1 | 0.8 | Action | 0.08 |
| R5 | No | 1 | 1 | 1 | 1 | 0.1 | 1 | 1 | 0.8 | Action | 0.08 |
| R6 | No | 1 | 1 | 1 | 1 | 1 | 1 | 1 | 0.8 | Joint | 0.8 |
| R7 | No | 1 | 1 | 1 | 1 | 0.1 | 1 | 1 | 0.8 | Action | 0.08 |
| R8A | Yes | 10 | 2 | 2 | 1 | 1 | 1 | 1 | 0.8 | Diagnosis | 32 |
| R8B | Yes | 10 | 2 | 2 | 1 | 1 | 1 | 1 | 0.8 | Action | 32 |
| R9 | No | 0.1 | 1 | 1 | 1 | 0.1 | 1 | 1 | 0.8 | Action | 0.008 |
| R10 | No | 0.1 | 1 | 1 | 1 | 1 | 1 | 1 | 0.8 | Action | 0.08 |
| R11 | No | 1 | 1 | 1 | 1 | 1 | 1 | 1 | 1 | Joint | 1 |
| R12 | Yes | 10 | 2 | 2 | 1 | 1 | 1 | 1 | 1 | Joint | 40 |
| R13 | No | 1 | 1 | 1 | 1 | 1 | 1 | 1 | 0.8 | Joint | 0.8 |
| R14 | Yes | 10 | 2 | 1 | 1 | 1 | 1 | 1 | 0.8 | Joint | 16 |
| R15 | No | 1 | 1 | 1 | 1 | 1 | 1 | 1 | 1 | Joint | 1 |
| R16 | No | 1 | 1 | 2 | 1 | 1 | 1 | 1 | 0.8 | Diagnostic | 1.6 |
| R17 | No | 1 | 1 | 2 | 1 | 1 | 1 | 1 | 0.8 | Joint | 1.6 |
| R18 | No | 1 | 1 | 1 | 1 | 1 | 1 | 1 | 1 | Joint | 1 |

**Basis for PSF assignments.** The baseline PSF levels in Table A4 were assigned by the author team using a structured, document-grounded expert-judgment process. Several members of the author team had prior research experience with Coyote Valley Dam operations and its Water Control Manual. Operational features of each rule-governed action were first identified from the rule content and operational context, and a common decision protocol developed through author consensus was then used to map those features to the corresponding SPAR-H levels. The operational features are therefore grounded in the source documents, while their translation into particular SPAR-H multipliers necessarily involves analyst judgment.

The following decision rules were applied consistently across the rule corpus. (i) Actions explicitly associated with emergency operations (R8A, R8B, R12, and R14) were assigned barely adequate Available Time (×10) and elevated Stressors (×2), reflecting the compressed decision conditions of emergency operation. Continuous monitoring actions without comparable time pressure (R9 and R10) were assigned extra Available Time (×0.1); other actions were assigned nominal Available Time and Stressors (×1). (ii) Complexity was elevated (×2) for actions involving interpretation or coordination across organizational or infrastructure boundaries (R1, R2, R3, R16, and R17) and for multi-element emergency tasks (R8A, R8B, and R12). Other actions, including the more procedurally direct emergency configuration in R14, were assigned nominal Complexity (×1). (iii) A favorable Procedures multiplier (×0.1) was assigned where the Water Control Manual provides relatively explicit procedural guidance for carrying out the action (R3, R4, R5, R7, and R9); other actions were assigned the nominal level (×1). (iv) Experience/Training, Ergonomics/HMI, and Fitness for Duty were held at nominal levels (×1) throughout, consistent with the assumption that operations are performed by trained and qualified personnel under normally functioning work conditions. (v) Work Processes were assigned a favorable multiplier (×0.8) where the action was judged to be embedded in an established operational or coordination workflow identified in the manual; otherwise the nominal level (×1) was used.

These rules provide a consistent basis for the baseline assignments but do not imply that the PSF levels are uniquely determined by the source documents. Because the mapping from operational context to SPAR-H categories necessarily involves analyst judgment, the robustness of these assignments is evaluated through the Monte Carlo sensitivity analysis in Appendix D.

**Table A5.** Summary of various rates and sources across the nodes in the Bayesian network in supporting material

| Blue node (label) | Best-fit component class | λ (h⁻¹) | P₁ₕ = 1–e^(–λ) | Source |
| --- | --- | --- | --- | --- |
| Potter Valley Powerhouse Tunnel | Power Plant | 8.68 × 10⁻⁸ | 8.68 × 10⁻⁸ | (National Renewable Energy Laboratory, 2023) |
| Coyote Dam | Large CFRD/arch dam | 3.20 × 10⁻⁸ | 3.20 × 10⁻⁸ | (Mitman, 2010) |
| Spillway Gate | Radial (Tainter) gate | 6.85 × 10⁻⁹ | 6.85 × 10⁻⁹ | (U.S. Bureau of Reclamation, 2019) |
| Drum Gate | Drum gate | 7.53 × 10⁻⁸ | 7.53 × 10⁻⁸ | (U.S. Army Corps of Engineers, 2017) |
| Plenum Tainter Valve | Large sluice/Tainter valve | 5.48 × 10⁻⁶ | 5.48 × 10⁻⁶ | (National Renewable Energy Laboratory, 2023) |
| Outlet Valve | Submerged butterfly/needle | 1.60 × 10⁻⁶ | 1.60 × 10⁻⁶ | (Idaho National Laboratory, 2009) |
| Outlet Conduit | Concrete conduit integrity | 1.00 × 10⁻⁶ | 1.00 × 10⁻⁶ | (OECD/NEA, 2003) |
| Intake Tower | Lightly reinforced intake tower | 5.00 × 10⁻⁸ | 5.00 × 10⁻⁸ | (Resatalab et al., 2021) |
| Power Plant | Turbine-generator availability | 8.68 × 10⁻⁸ | 8.68 × 10⁻⁸ | (National Renewable Energy Laboratory, 2023) |
| Tailrace Channel | Structural stability | 1.00 × 10⁻⁸ | 1.00 × 10⁻⁸ | (Lin et al., 2013) |
| Water-Level Sensor | Industrial pressure transducer | 2.00 × 10⁻⁷ | 2.00 × 10⁻⁷ | (Setra Systems, 2020) |
| Operation Program (software) | PLC-based control logic | 2.00 × 10⁻⁸ | 2.00 × 10⁻⁸ | (Onaifo et al., 2019) |
| Downstream Water Control | Software | 1.14 × 10⁻⁷ | 1.14 × 10⁻⁷ | (Adamo et al., 2020) |
| Radio / SCADA Link | Telemetry channel | 1.00 × 10⁻⁷ | 1.00 × 10⁻⁷ | (North American Electric Reliability Corporation, 2013) |
| Stream Gauge | Field sensor + telemetry | 2.00 × 10⁻⁷ | 2.00 × 10⁻⁷ | (Setra Systems, 2020) |

**Basis for the one-hour exposure interval.** The component reliability sources in Table A5 report failure rates λ on an hourly basis. To convert these rates into probabilities for Bayesian network parameterization, we use a common exposure interval of t=1 hour, such that P₁ₕ = 1–e^(–λ). This choice is also consistent with the relatively short operational timescale of monitoring and control at Coyote Valley Dam. Water-control procedures use regularly timed gage and flow observations, allow more frequent computations during flood operations when needed, and constrain changes in flood-control releases on an hourly basis; several individual operational steps also occur on sub-hourly timescales. The one-hour interval is therefore treated as a common reference exposure window rather than as the exact duration of every operational task or complete operating-mode transition.

**Table A6.** Baseline reference HEPs and posterior probabilities of rule violations with corresponding Ratio of Variation (RoV) values.

| **Rule No.** | **Baseline HEP** | **Posterior** | **ROV** |
| --- | --- | --- | --- |
| R1 | 0.016 | 0.22642511 | 13.15156938 |
| R2 | 2.50E-05 | 0.001049196 | 40.967836 |
| R3 | 2.60E-07 | 2.60E-04 | 999.5714231 |
| R4 | 8.00E-05 | 3.24E-04 | 3.04479175 |
| R5 | 8.00E-05 | 0.005861211 | 72.26513375 |
| R6 | 6.40E-06 | 2.65E-04 | 40.4083125 |
| R7 | 8.00E-05 | 0.006975763 | 86.1970325 |
| R8A | 0.24 | 0.82237311 | 2.426554625 |
| R8B | 0.031 | 0.03121449 | 0.006919032 |
| R9 | 8.00E-06 | 2.66E-04 | 32.2839725 |
| R10 | 8.00E-05 | 3.24E-04 | 3.04479175 |
| R11 | 1.00E-05 | 2.68E-04 | 25.787029 |
| R12 | 0.011 | 0.014298728 | 0.299884364 |
| R13 | 6.40E-06 | 0.005735941 | 895.2408438 |
| R14 | 0.002 | 0.007710768 | 2.85538415 |
| R15 | 1.00E-05 | 2.00E-05 | 0.99999 |
| R16 | 0.016 | 0.019142643 | 0.196415188 |
| R17 | 2.50E-05 | 2.80E-04 | 10.2112508 |
| R18 | 1.00E-05 | 2.00E-05 | 0.99999 |

**Appendix B**

**Box B1: Institutional Grammar coding of rules**

**Worked Example 1**

Rule statement (R2): *The Corps will establish, and transmit to the City, dam flow release schedules at Coyote Dam for a weekly, daily, or other period as needed.*

**ATTRIBUTE:**	[The Corps]

**DEONTIC:**	[will]

**AIM:**	[establish and transmit]

**OBJECT:**	[dam flow release schedules]

**CONTEXT:**	[at Coyote Dam; for a weekly, daily, or other period as needed]

**Worked Example 2**

Rule statement (R17): *Coyote Valley operators will provide routine and emergency instructions and support for Water Control Data Systems (WCDS)*

**ATTRIBUTE:**	[Coyote Valley operators]

**DEONTIC:**	[will]

**AIM:**	[provide]

**OBJECT:**	[Water Control Data Systems (WCDS)]

**CONTEXT:**	[in both routine and emergency instruction cases)]

**Worked Example 3**

Rule statement (R15): *SCWA diverts municipal water at its pumping stations in the vicinity of Wohler Bridge below Healdsburg.*

**ATTRIBUTE:**	[SCWA]

**DEONTIC:**	[will]

**AIM:**	[divert]

**OBJECT:**	[municipal water]

**CONTEXT:**	[at its pumping stations in the vicinity of Wohler Bridge below Healdsburg]

**Box B2: From rule to hazard, safety constraint, and control structure**

**Worked Example 1**

Rule statement (R14): “When the dam and power plant facilities are operating in the flood control mode, the plenum tainter valve will be fully open, the turbines and bypass valves will be fully closed, and the control gates will be opened as necessary to pass the required water release.”

**HAZARD IMPLICATED:** Unsafe flood-control release operation, potentially contributing to **Coyote Valley Dam failure or downstream community impacts.**

**SAFETY CONSTRAINT:** In flood-control mode, reservoir releases must be routed through the intended flood-control pathway so that the required discharge is passed safely without relying on the power-generation pathway.

**CONTROL ACTION:** Reservoir operators must **fully open the plenum tainter valve, keep the turbines and bypass valves fully closed,** and **open the control gates as necessary to pass the required flood release.**

**CONTROLLED PROCESS: The controlled objects are the plenum tainter valve, turbines, bypass valves, and control gates; the controlled process is the routing and discharge of water releases from Coyote Dam under flood-control mode.**

**FEEDBACK REQUIRED:** Information on (1) whether the system is in flood-control mode, (2) the required release level, and (3) the current status of the plenum tainter valve, turbines, bypass valves, and control gates.

**Worked Example 2**

Rule statement (R13): “*When the dam and power plant facilities are operating in the power generation mode, the control gates (three service slide gates and three emergency slide gates in the intake tower) will be fully open, the plenum tainter valve will be fully closed, and the turbines and bypass valves will be opened as necessary to pass the required water release”*

**HAZARD IMPLICATED:** Unsafe power-generation release operation, potentially contributing to **downstream community impacts.**

**SAFETY CONSTRAINT: In power generation mode,** reservoir releases must be routed through the intended power-generation pathway so that the required discharge is passed safely without relying on the flood-control pathway.

**CONTROL ACTION:** Reservoir operators must **fully open the control gates, keep the plenum tainter valve fully closed,** and **open the turbines and bypass valves as necessary to pass the required water release.**

**CONTROLLED PROCESS: The controlled objects are the control gates, plenum tainter valve, turbines, and bypass valves; the controlled process is the routing and discharge of water releases from Coyote Dam under power generation mode.**

**FEEDBACK REQUIRED:** Information on (1) whether the system is in power generation mode, (2) the required release level, and (3) the current status of the **control gates, plenum tainter valve, turbines, and bypass valves.**

**Worked Example 3**

Rule statement (R10): “*The city will be responsible for maintaining a minimum dissolved oxygen level of 7.0 mg/l at the existing U.S.G.S. stream gage immediately downstream of the proposed tailrace”*

**HAZARD IMPLICATED: I**mproper operation of the outlet valve causing dissolved oxygen levels to fall under 7.0 mg/l at the U.S.G.S stream gage downstream of the tailrace, potentially contributing to degrading water quality **which impacts downstream community.**

**SAFETY CONSTRAINT: The outlet valve must be operated to maintain a dissolved oxygen level of at least 7.0 mg/l at all times.**

**CONTROL ACTION:** The city must adjust the outlet valve when dissolved oxygen levels are under 7.0 mg/l at U.S.G.S stream gage.

**CONTROLLED PROCESS: The controlled objects are outlet valve and water control data systems, and the controlled process is water discharge from the outlet valve to the U.S.G.S strean gage.**

**FEEDBACK REQUIRED:** Information on (1) dissolved oxygen level at U.S.G.S strean gage, (2) the current status of the outlet valve.

**Box B3: Deriving an ICA from a governing rule**

**Worked Example 1**

Rule statement (R14): “When the dam and power plant facilities are operating in the flood control mode, the plenum tainter valve will be fully open, the turbines and bypass valves will be fully closed, and the control gates will be opened as necessary to pass the required water release.”

**Control relation:**
Reservoir operators implement and maintain the flood-control configuration of the plenum tainter valve, turbines, bypass valves, and control gates.

**ICA example:**
A required control action is not provided if operators fail to fully open the plenum tainter valve or fail to open the control gates as necessary when flood-control mode is active. An unsafe control action is provided if turbines or bypass valves remain open when they should be fully closed under flood-control mode.

**Worked Example 2**

Rule statement (R13): “*When the dam and power plant facilities are operating in the power generation mode, the control gates (three service slide gates and three emergency slide gates in the intake tower) will be fully open, the plenum tainter valve will be fully closed, and the turbines and bypass valves will be opened as necessary to pass the required water release”*

**Control relation:**

Reservoir operators implement and maintain the power generation configuration of the control gates, plenum tainter valve, turbines, and bypass valves.

**ICA example:**
A required control action is not provided if operators fail to fully open the control gates or fail to open the turbines or bypass valves as necessary when power generation mode is active. An unsafe control action is provided if plenum tainter valve remain open when it should be fully closed under power generation mode.

**Worked Example 3**

Rule statement (R10): “*The city will be responsible for maintaining a minimum dissolved oxygen level of 7.0 mg/l at the existing U.S.G.S. stream gage immediately downstream of the proposed tailrace”*

**Control relation:**

The city maintains a minimum dissolved oxygen level of 7.0 mg/l measured at the U.S.G.S stream gage.

**ICA example:**

A required control action is not provided if the water control data systems signal that dissolved oxygen level is under 7.0 mg/l and the city fails to adjust the outlet valve to return it to the desirable range. An unsafe control action is provided if the city takes an action that causes the dissolved oxygen level to fall under 7.0 mg/l at the U.S.G.S stream gage (outlet valve is too wide/too restricted).

**Box B4: SPAR-H application**

**Worked Example 1**

(R1): *All water releases from Coyote Dam may fail to be determined, controlled, and directed by the Corps.*

**Category:** Diagnosis

**Performance Shaping Factors (SPF):**

Emergency: No

Available time:

Rationale: Standard operational scenarios with adequate time for task completion

Nominal time $\to$ Multiplier $= 1$

Stressors:

Rationale: Non-emergency scenarios with standard operational stress levels

Nominal stress $\to$ Multiplier $= 1$

Complexity:

Rationale: Complex coordination tasks (water release determination)

Moderately complex $\to$ Multiplier $= 2$

Experience/training:

Rationale: Adequate experience and training is sufficient for this routine task’s demands.

Nominal $\to$ Multiplier $= 1$

Procedures:

Rationale: Standard operational procedures

Nominal $\to$ Multiplier $= 1$

Ergonomics/HMI:

Instruments are functional and accessible with no HMI deficiencies affecting operator’s performance.

Nominal $\to$ Multiplier $= 1$

Fitness for duty:

Rational: Operator is fit for duty with no conditions affecting performance.

Nominal $\to$ Multiplier $= 1$

Work process:

Rationale: Inter-agency coordination scenarios involving USACE-Ukiah communication and established protocols

Good $\to$ Multiplier $= 0.8$

**PSF Composite multiplier** $\boldsymbol{=1 \times1\times2\times1\times1\times1\times1\times0.8=1.6}$

**Human Error Probabilities (HEP):**

$NHEP= 1.0\times10⁻^{2}$ (diangnosis)

$HEP= NHEP\times PSF_{composite}=1.0\times{10}^{-2}\times1.6=0.016$ $\leftarrow$ Prior probability P(C)

**Worked Example 2**

(R5): *The Corps may fail to get notified immediately of any revisions to this program.*

**Category:** Action

**Performance Shaping Factors (SPF):**

Emergency: No

Available time:

Rationale: Standard operational scenarios with adequate time for task completion

Nominal time $\to$ Multiplier $= 1$

Stressors:

Rationale: Non-emergency scenarios with standard operational stress levels

Nominal stress $\to$ Multiplier $= 1$

Complexity:

Rationale: Standard operational tasks with routine procedural requirements

Moderately complex $\to$ Multiplier $= 1$

Experience/training:

Rationale: Adequate experience and training is sufficient for this routine task’s demands.

Nominal $\to$ Multiplier $= 1$

Procedures:

Rationale: Information-intensive task with structured procedural guidance

Nominal $\to$ Multiplier $= 0.1$

Ergonomics/HMI:

Instruments are functional and accessible with no HMI deficiencies affecting operator’s performance.

Nominal $\to$ Multiplier $= 1$

Fitness for duty:

Rational: Operator is fit for duty with no conditions affecting performance.

Nominal $\to$ Multiplier $= 1$

Work process:

Rationale: Inter-agency coordination scenarios involving USACE-Ukiah communication and established protocols

Good $\to$ Multiplier $= 0.8$

**PSF Composite multiplier** $\boldsymbol{=1 \times1\times1\times1\times0.1\times1\times1\times0.8=0.08}$

**Human Error Probabilities (HEP):**

$NHEP= 1.0\times{10}^{-3}$ (action)

$HEP= NHEP\times PSF_{composite}=1.0\times{10}^{-3}\times0.08=8\times{10}^{-5}$ $\leftarrow$ Prior probability P(C)

**Worked Example 3**

(R15): *SCWA may fail to divert municipal water at its pumping stations in the vicinity of Wohler Bridge below Healdsburg.*

**Category:** Joint (Diagnosis + Action)

**Performance Shaping Factors (SPF):**

Emergency: No

Available time:

Rationale: Standard operational scenarios with adequate time for task completion

Nominal time $\to$ Multiplier $= 1$

Stressors:

Rationale: Non-emergency scenarios with standard operational stress levels

Nominal stress $\to$ Multiplier $= 1$

Complexity:

Rationale: Standard operational tasks with routine procedural requirements

Moderately complex $\to$ Multiplier $= 1$

Experience/training:

Rationale: Adequate experience and training is sufficient for this routine task’s demands.

Nominal $\to$ Multiplier $= 1$

Procedures:

Rationale: Standard operational procedures

Nominal $\to$ Multiplier $= 1$

Ergonomics/HMI:

Instruments are functional and accessible with no HMI deficiencies affecting operator’s performance.

Nominal $\to$ Multiplier $= 1$

Fitness for duty:

Rational: Operator is fit for duty with no conditions affecting performance.

Nominal $\to$ Multiplier $= 1$

Work process:

Rationale: Single-agency tasks with standard coordination requirements

Nominal work process $\to$ Multiplier $= 1$

**PSF Composite multiplier** $\boldsymbol{=1 \times1\times1\times1\times1\times1\times1\times1=1}$

**Human Error Probabilities (HEP):**

${HEP}_{action}^{R_{15}}= NHEP\times PSF_{composite}=1.0\times{10}^{-3}\times1=1\times{10}^{-3}$

$${HEP}_{diagnosis}^{R_{15}}= NHEP\times PSF_{composite}=1.0\times{10}^{-2}\times1=1\times{10}^{-2}$$

$${HEP}_{joint}^{R_{15}}= {HEP}_{action}^{R_{15}}\times{HEP}_{diagnosis}^{R_{15}}$$

$${HEP}_{joint (AND)}^{R_{15}}= \left( 1\times{10}^{-3} \right)\times\left( 1\times{10}^{-2} \right)=1\times{10}^{-5}$$

**Box B5: Bayesian Network Parameterization of rule, actor, and infrastructure nodes**

**Worked Example 1**

(R1): *All water releases from Coyote Dam may fail to be determined, controlled, and directed by the Corps.*

$HEP=P\left( R1 \right)=0.016$ $\leftarrow$ Prior probability

SPAR-H gives independent failure probability of each rule, and it is assumed that each rule contributes equally to the overall error probability. Therefore, the probability of error in an actor is the average of the failure probabilities of the rules it has to follow:

$$P_{error}\left( {USACE}_{8} \right)=\frac{1}{n_{k}}\sum_{i=1}^{n_{k}} {HEP}_{t}^{R_{i}}=\frac{R1+R2+R7+R8A+R8B+R12+R13+R14}{8}$$

$$=\frac{0.016+\left( 2.50\times{10}^{-5} \right)+\left( 8.00{\times10}^{-5} \right)+0.24+0.031+0.011+\left( 6.40{\times10}^{-6} \right)+0.002}{8}\approx0.03751$$

$$P\left( {Coyote Dam}_{Failure} \right) =1-e^{-\lambda t}$$

$= 1-e^{-3.20 \times{10}^{-8(1)}}$ $\to\lambda$ is sourced from (Mitman, 2011)

$= 3.20 \times10⁻⁸$

Posterior probabilities are calculated using GeNIe (Posterior probability for R1$=0.22642511)$

$$RoV\left( X_{i} \right)=\frac{P\left( X_{i} \mid E \right)-P\left( X_{i} \right)}{P\left( X_{i} \right)}=\frac{0.2264251-0.016}{0.016}=13.15$$

**Worked Example 2**

(R5): *The Corps may fail to get notified immediately of any revisions to this program.*

$HEP=P\left( R5 \right)=8\times{10}^{-5}$ $\leftarrow$ Prior probability

SPAR-H gives independent failure probability of each rule, and it is assumed that each rule contributes equally to the overall error probability. Therefore, the probability of error in an actor is the average of the failure probabilities of the rules it has to follow:

$P_{error}\left( {Ukiah City}_{11} \right)=\frac{1}{n_{k}}\sum_{i=1}^{n_{k}} {HEP}_{t}^{R_{i}}=\frac{R7+R5+R6+R14+R13+R3+R17+R4+R9+R10+R11+R12}{12}$

$$=\frac{\left( 8.00{\times10}^{-5} \right)+\left( 8.00{\times10}^{-5} \right)+\left( 6.4{\times10}^{-6} \right)+0.002+\left( 6.4{\times10}^{-6} \right)+\left( 2.6{\times10}^{-7} \right)+\left( 2.5{\times10}^{-5} \right)+\left( 8.00{\times10}^{-5} \right)+\left( 8.00{\times10}^{-6} \right)+\left( 8.00{\times10}^{-5} \right)+\left( 1.00{\times10}^{-5} \right)+0.011}{12}$$

$$\approx1.114{\times10}^{-3}$$

$$P\left( {Coyote Dam}_{Failure} \right) =1-e^{-\lambda t}$$

$= 1-e^{-3.20 \times{10}^{-8(1)}}$ $\to\lambda$ is sourced from (Mitman, 2011)

$= 3.20 \times10⁻⁸$

Posterior probabilities are calculated using GeNIe (Posterior probability for R5$=0.005861211)$

$$RoV\left( X_{i} \right)=\frac{P\left( X_{i} \mid E \right)-P\left( X_{i} \right)}{P\left( X_{i} \right)}=\frac{0.005861-\left( 8\times{10}^{-5} \right)}{8\times{10}^{-5}}=72.26$$

**Worked Example 3**

(R15): *SCWA may fail to divert municipal water at its pumping stations in the vicinity of Wohler Bridge below Healdsburg.*

$HEP=P\left( R15 \right)=1\times{10}^{-5}$ $\leftarrow$ Prior probability

SPAR-H gives independent failure probability of each rule, and it is assumed that each rule contributes equally to the overall error probability. Therefore, the probability of error in an actor is the average of the failure probabilities of the rules it has to follow:

$P_{error}\left( {SCWA}_{1} \right)=1\times{10}^{-5}$ (Governs one rule only)

$$P\left( {Downstream communities}_{Failure} \right) =1-e^{-\lambda t}$$

$= 1-e^{-1.14 \times10⁻⁷(1)}$ $\to\lambda$ is sourced from (Adamo et al., 2020)

$= 1.14 \times10⁻⁷$

Posterior probabilities are calculated using GeNIe (Posterior probability for R15$=2\times{10}^{-5})$

$$RoV\left( X_{i} \right)=\frac{P\left( X_{i} \mid E \right)-P\left( X_{i} \right)}{P\left( X_{i} \right)}=\frac{\left( 2\times{10}^{-5} \right)-\left( 1\times{10}^{-5} \right)}{1\times{10}^{-5}}=0.99999$$

**Appendix C**

**Model validation**

To provide reasonable confidence in the proposed method, a three‑axiom‑based approach is used for partial validation of the quantitative analysis. The axioms check that the Bayesian Network’s probabilistic influence behaves logically (Jones et al., 2010; Liu & Liu, 2019). In the context, the model stratifies three axioms. **Axiom 1-**When the prior failure probability of a parent increases (or decreases), the posterior failure probability of the child should increase (or decrease) correspondingly. Example: In Figure 4, the Outlet Valve and R15 as parents of Wohler Bridge failure. If the R15 failure probability increases from ${1.0e}^{-5}$ to ${0.5e}^{-1}$, Wohler Bridge failure should move from ${1.6e}^{-1}$ to ${2.0e}^{-1}$. **Axiom 2** - Changes in different parents should push the child’s probability in a consistent direction. Example: In Figure 4, increasing either Outlet Valve (from ${1.6e}^{-1}$ → ${3.5e}^{-1}$) or R15 (from ${1.0e}^{-5}$ → ${0.5e}^{-1}$) each increases Wohler Bridge failure. Conversely, decreasing either parent reduces the bridge failure probability by a commensurate amount. **Axiom 3** - The total influence from a set of parents should exceed the influence of any proper subset. Example: In Figure 4, increasing both Outlet Valve and R15 together should produce a larger change in Wohler Bridge failure (from ${1.6e}^{-1}$ → ${3.8e}^{-1}$) than changing R15 alone.

**Figure C1.** Model Validation showcasing the example of Rule R15 and the Outlet Valve as parent nodes of Wohler Bridge. The figure shows that nodes are consistent with three validation axioms.

**Appendix D**

**Sensitivity Analysis**

**D.1 Motivation & Scope**

The Bayesian model presented in Section 2.5 relies on Performance Shaping Factor (PSF) assignments to parameterize Human Error Probabilities (HEPs) for each rule-governed action via the SPAR-H method. Because PSF selection involves expert judgment, different analysts assessing the same rule-governed action may arrive at different PSF levels, producing different HEPs and potentially different risk conclusions. This appendix reports a Monte Carlo sensitivity analysis designed to evaluate whether the framework's principal findings, particularly the identification of hidden logical risk rules and the non-trivial nature of systemic risk from logical interdependencies, are robust to plausible variation in PSF assignments. The analysis varies the SPAR-H-derived priors (HEPs and actor failure probabilities) that enter the Bayesian network as input parameters. Infrastructure component failure rates, which are drawn from published reliability data (Appendix Table A5), are held fixed. This focus on human/rule parameters is deliberate. The PSF assignments involve analyst judgment and therefore represent a direct source of epistemic uncertainty introduced during model parameterization. Infrastructure component failure rates, in contrast, are taken from published reliability sources and are held fixed here so that the analysis can isolate the effects of uncertainty in the SPAR-H-derived parameters. Holding these rates fixed does not imply that hardware reliability is known without uncertainty; rather, uncertainty in the component failure rates is outside the scope of the present sensitivity analysis. Future applications could extend the analysis by jointly varying human/rule and hardware reliability parameters. The Bayesian network structure (nodes, edges) also remains fixed, as it is derived from the STAMP-based control architecture rather than from subjective assessment.

**D.2** **Sampling** **Strategy**

For each of the 19 rule-governed actions, eight PSFs are assigned discrete multiplier levels drawn from the SPAR-H methodology (Table D1). Since in this study we focus on human error in decision making with assumption they have adequate training, three PSFs, Experience/ Training, Ergonomics/HMI, and Fitness for Duty, are held fixed at their baseline values throughout the sensitivity analysis. Four PSFs, Available Time, Complexity, Procedures, and Work Processes, are independently varied by one level above or below their baseline assignment on the ordered PSF scale. For example, if a rule's baseline Complexity is assessed as "Moderately Complex" (multiplier = 2), the sensitivity analysis samples from {Nominal (1), Moderately Complex (2), Highly Complex (5)}, but does not permit "Obvious Diagnosis" (0.1), which would contradict the operational nature of that rule. The eighth PSF, Stress/Stressors, is derived as a composite condition from the sampled Available Time and Complexity values using the product AT × C: AT × C ≤ 2 yields Stressors = 1 (nominal), 2 < AT × C ≤ 20 yields Stressors = 2 (high), and AT × C > 20 yields Stressors = 5 (extreme). This formulation reflects the domain understanding that operator stress arises from the interaction of time pressure and task complexity, and reproduces all 19 baseline Stressor assignments exactly. Two additional constraints preserve scenario coherence: emergency rules (R8A, R8B, R12, R14) are restricted to Available Time multipliers of 10 or greater, and non-emergency rules are restricted to Available Time multipliers of 1 or less. A triangular (peaked) sampling distribution assigns 50% probability to the baseline level and 25% to each adjacent level (for three allowed levels), reflecting that the baseline represents the best expert judgment rather than one of several equally plausible alternatives. Results are also compared against uniform sampling to assess sensitivity to the distributional assumption.

**Table** **D1.** SPAR-H Performance Shaping Factor levels used in the sensitivity analysis.

| **Performance Shaping Factor** | **Levels (Multipliers)** |
| --- | --- |
| Available Time | 0.01, 0.1, 1, 10, 50 |
| Stress/Stressors | 1, 2, 5 |
| Complexity | 0.1, 1, 2, 5 |
| Experience/Training | 0.5, 1, 3 |
| Procedures | 0.1, 1, 5, 20, 50 |
| Ergonomic | 0.5, 1, 10, 50 |
| Fitness for Duty | 1, 5 |
| Work Processes | 0.8, 1, 5 |

**D.3 Monte Carlo Procedure**

The analysis proceeded over 1,000 iterations. In each iteration:

I. Four PSF levels (Available Time, Complexity, Procedures, Work Processes) were sampled from the rule-specific allowed ranges described in D.2 using a triangular distribution centered on the baseline; Stressors was derived from the sampled Available Time and Complexity; Experience, Ergonomics, and Fitness for Duty were held at baseline values
II. Composite PSF multipliers and HEPs were recomputed following Eqs.1-4
III. Actor priors were recalculated as the mean HEP of governed rules (Eq. 5)
IV. All Bayesian network conditional probability tables containing HEP-derived values were updated, while infrastructure failure rates remained fixed
V. Forward inference yielded end-node failure probabilities
VI. Backward inference, conditioned on Coyote Valley Dam failure, yielded posterior probabilities and Ratio of Variation (RoV) values for each rule
VII. Each rule was classified into one of the four risk quadrants defined in Section 3.4.

**D.4** **End-Node** **Failure** **Probability** **Distributions**

Forward inference across all iterations produced distributions of failure probability for each end node (Fig. D1). Under the triangular distribution, the mean failure probability for Coyote Valley Dam was 34.0% (95% distribution interval: [1.5%, 82.8%]), with a median of 25.6%. For Downstream Communities, the mean was 23.5% (95% interval: [2.0%, 66.5%]), with a median of 16.8%. Under uniform sampling, these values were moderately higher (Coyote Dam mean 41.6%, Downstream Communities mean 31.4%), confirming that the peaked distribution produces more conservative estimates by favoring the baseline. The distributions remain wide, reflecting the multiplicative structure of SPAR-H, but are substantially narrower than in preliminary analyses that varied all eight PSFs independently, demonstrating the importance of fixing stable organizational factors and deriving stress as a composite condition.

Despite this variability in absolute magnitudes, the finding that logical interdependencies generate non-trivial systemic risk is robust. Under triangular sampling, Coyote Dam failure probability exceeded 1% in 97.2% of iterations and exceeded 5% in 84.7%. For Downstream Communities, failure probability exceeded 1% in 97.7% of iterations and exceeded 3% in 90.0%. The baseline values reported in Section 3.1 (7% and 3%) fall within the central portion of these distributions.

A scenario comparison (Fig. D5) further illustrates how end-node probabilities change across alternative deterministic PSF profiles used for sensitivity analysis. Under uniformly optimistic PSF assumptions, failure probabilities dropped to near zero. Under the nominal sensitivity profile, Coyote Dam failure probability was 5.0% and Downstream Communities was 2.1%. Pessimistic and worst-case scenarios produced near-certain failure. These scenario profiles are sensitivity cases and are distinct from the baseline model reported in Section 3.1.

**Figure D1**. Distribution of end-node failure probabilities across 1,000 Monte Carlo iterations using triangular PSF sampling. Each panel shows the histogram of P(Failure) for one end node: Coyote Valley Dam (left), Downstream Communities (center), and Potter Valley Powerhouse Tunnel (right). Dashed red and dotted orange lines indicate the mean and median, respectively. Three PSFs (Experience, Ergonomics, Fitness for Duty) are held at baseline; four (Available Time, Complexity, Procedures, Work Processes) are varied ±1 adjacent SPAR-H level with peaked weighting; Stressors is derived from AT × Complexity.

**D.5 Quadrant Robustness**

The central analytical question is whether the risk quadrant classifications remain stable when PSF assignments vary. For each iteration, RoV was recomputed using the HEP as the reference probability (consistent with Eq. 8), and each rule was classified into one of the four quadrants using the thresholds defined in Section 3.4 (HEP ≥ 1 × 10⁻³ and RoV ≥ 5). Fig. D2 presents the results as a stacked bar chart in which each bar represents one rule, divided into four colored segments corresponding to the percentage of iterations in which that rule was assigned to each quadrant. Rules are grouped by their baseline quadrant assignment, and the baseline segment is marked with diagonal hatching. A red triangle indicates rules whose most frequently assigned quadrant differs from their baseline assignment.

**Figure D2**. Quadrant assignment robustness across 1,000 Monte Carlo iterations with four PSFs varied by one adjacent SPAR-H level (Available Time, Complexity, Procedures, Work Processes), three held fixed (Experience, Ergonomics, Fitness for Duty), and Stressors derived from AT × Complexity. Each bar represents one rule and is divided into four segments corresponding to the percentage of iterations in which that rule was classified into each quadrant: Critical Systemic Risk (red), Hidden Logical Risk (orange), Localized Execution Risk (blue), and Minimal Risk (gray). Rules are grouped by their baseline quadrant assignment, and the baseline segment is marked with diagonal hatching. A red triangle indicates rules whose most frequently assigned quadrant differs from their baseline.

Three principal findings emerge. First, the nine Hidden Logical Risk rules identified in Section 3.4 (R2, R3, R5, R6, R7, R9, R11, R13, R17) maintain their classification with high stability under PSF perturbation: R13 remains in the Hidden Logical Risk quadrant in 98.0% of iterations, R3 in 90.2%, R5 in 89.6%, R7 in 88.8%, R2 in 87.7%, R6 in 85.2%, R11 in 76.9%, R9 in 75.0%, and R17 in 70.2%. These results indicate that the Hidden Logical Risk classifications are generally stable under the tested PSF perturbations rather than being highly sensitive to the baseline PSF selections.

Second, the Localized Execution Risk rules (R8A, R8B, R12, R14, R16) are predominantly stable, with R8B at 97.5%, R8A at 95.3%, R16 at 84.6%, and R12 at 81.0% in their baseline quadrant. R14 shows substantial variability at 45.1%, though its most frequent assignment remains Localized Execution Risk. Their combination of high baseline error propensity and lower posterior amplification is, with the exception of R14, robust to PSF variation.

Third, R4 and R10 sit in the Minimal Risk quadrant at baseline because their RoV values (both 3.0) fall just below the threshold of 5. Under PSF perturbation, however, both rules more frequently classify as Hidden Logical Risk (52.6% and 52.7% of iterations respectively) than as Minimal Risk (37.7% and 33.8%). This boundary behavior shows that the line between Minimal and Hidden Logical Risk is sensitive to PSF assessment for rules whose RoV sits near the threshold, and that the set of structurally important rules may extend slightly beyond the nine identified at baseline.

**D.6** **Influential** **Performance** **Shaping** **Factors** **and** **Rules**

A correlation analysis identified which rules and PSFs exert the strongest influence on Coyote Dam failure probability across Monte Carlo iterations. At the rule level (Fig. D3), R8A (emergency advisory protocol, r = 0.97) dominates, reflecting its high baseline HEP and direct connection to dam failure. R1 (release authority, r = 0.17) shows moderate influence. The remaining rules show weak correlations (|r| < 0.08), consistent with the structural finding that dam failure risk is concentrated in a small number of critical pathways.

**Figure D3.** Tornado diagram showing the Pearson correlation between each rule's HEP (varied across Monte Carlo iterations) and the probability of Coyote Valley Dam failure. Rules are ranked by absolute correlation strength. Emergency rules are shown in red; normal-operation rules in blue. R8A (emergency advisory protocol) dominates with r = 0.97, reflecting its high baseline HEP and direct structural connection to dam failure.

At the PSF level (Fig. D4), Stressors (r ≈ 0.35) and Available Time (r ≈ 0.29) were the most influential factors, followed by Complexity (r ≈ 0.14) and Procedures (r ≈ 0.13). Work Processes showed negligible influence (r ≈ 0.01). Experience, Ergonomics, and Fitness for Duty were held fixed at their baseline values, so their sensitivity was not assessed in this analysis. The dominance of Stressors reflects its derivation from the product of Available Time and Complexity, which amplifies their joint effect on emergency rules. These findings suggest that operational factors governing time pressure and task complexity, together with procedural quality, would most effectively reduce both the magnitude and the uncertainty of systemic risk estimates.

**Figure D4.** PSF-level tornado diagram showing the correlation between each Performance Shaping Factor (aggregated across all rules) and Coyote Valley Dam failure probability. Stressors (derived from Available Time × Complexity) and Available Time exhibit the strongest influence. Experience, Ergonomics, and Fitness for Duty were held fixed at baseline values throughout the analysis and therefore were not included in the sensitivity assessment.

**D.7** **Summary**

The sensitivity analysis demonstrates that while absolute failure probabilities are sensitive to SPAR-H PSF assignments (as expected given the method's multiplicative structure), the framework's principal qualitative findings are robust to plausible analyst disagreement. The identification of Hidden Logical Risk rules (R2, R3, R5, R6, R7, R9, R11, R13, R17) is stable, with quadrant assignment maintained in 70.2% to 98.0% of iterations. No Hidden Logical Risk rule shifts predominantly to the Minimal Risk quadrant in the modeled perturbation range. The analysis also reveals that R4 and R10, which sit in Minimal Risk at baseline because their RoV values fall just below the threshold of 5, more often reclassify as Hidden Logical Risk under perturbation, indicating that the set of structurally important rules may extend slightly beyond the nine identified at baseline. A comparison of uniform and triangular sampling distributions confirms that results are qualitatively consistent across distributional assumptions, with the triangular distribution producing moderately lower mean failure probabilities (Coyote Dam: 34.0% vs. 41.6%; Downstream Communities: 23.5% vs. 31.4%) that better reflect the baseline as the best expert judgment.

**Figure D5.** Scenario comparison of end-node failure probabilities under four deterministic PSF profiles used for sensitivity analysis: optimistic (PSFs at favorable levels), nominal (PSFs at nominal levels), pessimistic (degraded PSFs), and worst case (PSFs at maximum adverse levels), with emergency and non-emergency constraints applied. The vertical axis uses a logarithmic scale. These deterministic sensitivity profiles are distinct from the rule-specific baseline parameterization reported in the main text.

**Figure D6.** Comparison of end-node failure probability distributions under uniform and triangular PSF sampling. Blue histograms show uniform sampling (equal weight on all allowed levels); coral histograms show triangular sampling (50% weight on baseline, 25% on each adjacent level). Dashed vertical lines indicate means. The triangular distribution produces lower means, reflecting the baseline as best expert judgment.

**References**

Adamo, N., Al-Ansari, N., Sissakian, V., Laue, J., & Knutsson, S. (2020). Dam Safety: Hazards Created by Human Failings and Actions. *Journal of Earth Sciences and Geotechnical Engineering*, 65–107. https://doi.org/10.47260/JESGE/1113

Idaho National Laboratory. (2009). *Independent Review of Mitigating System Performance Indicator Reporting in the EPIX Database*. http://www.inl.gov

Jeffrey T. Mitman. (2010). *Generic Failure Rate Evaluation for Jocassee Dam*.

Jones, B., Jenkinson, I., Yang, Z., & Wang, J. (2010). The use of Bayesian network modelling for maintenance planning in a manufacturing industry. *Reliability Engineering & System Safety*, *95*(3), 267-277.

Lin, P., Zhou, Y., Liu, H., & Wang, C. (2013). Reinforcement design and stability analysis for large-span tailrace bifurcated tunnels with irregular geometry. *Tunnelling and Underground Space Technology*, *38*, 189–204.

Liu, Z., & Liu, Y. (2019). A Bayesian network based method for reliability analysis of subsea blowout preventer control system. *Journal of Loss Prevention in the Process Industries*, *59*, 44-53.

*National Renewable Energy Laboratory (NREL) (2023) Renewable Energy Data Book. - References - Scientific Research Publishing*. (n.d.). Retrieved April 22, 2026, from https://www.scirp.org/reference/referencespapers?referenceid=3884056

North American Electric Reliability Corporation. (2013). *SCADA failure resulting in loss of monitoring function (Lessons Learned 20130203)*. https://www.nerc.com/globalassets/programs/event-analysis/lessons-learned/ll20130203_scada_failure_resulting_in_loss_of_monitoring_function.pdf

OECD/NEA. (2003). *NUCLEAR ENERGY AGENCY COMMITTEE ON THE SAFETY OF NUCLEAR INSTALLATIONS ICDE PROJECT REPORT: COLLECTION AND ANALYSIS OF COMMON-CAUSE FAILURE OF CHECK VALVES*.

Onaifo, F., Okandeji, A. A., Folorunsho, O., Essien, U. E., Oyedeji, A. O., & Abolade, O. R. (2019). Comparison of the reliability of programmable logic controller and electromagnetic relay control in industrial production line. *Nigerian Journal of Technology*, *38*(4), 1030–1035. https://doi.org/10.4314/NJT.V38I4.28

Resatalab, S., Ahmadi, M. T., Alembagheri, M., & Kamboozia, N. (2021). *Effective Parameters on Seismic Response of Intake Towers, Including Reservoir, Foundation and Dam Interactions*. 24–27.

Setra Systems. (2020). *The Cost of a Failed Sensor*. https://www.setra.com/blog/the-cost-of-a-failed-sensor

U.S. Army Corps of Engineers. (2017). *Risk assessments for gates other than radial gates—Drum gates (Best Practices, Chap. G-2).* https://www.usbr.gov/ssle/damsafety/risk/BestPractices/Presentations/G2-RiskAssessmentOfGatesOtherThanRadialGatesPP.pdf

U.S. Bureau of Reclamation. (2019). *Failure of radial (Tainter) gates under normal operational conditions (Best Practices, Chap. G-1)*. https://www.usbr.gov/damsafety/risk/BestPractices/Chapters/G1-FailureOfRadial-Tainter-GatesUnderNormalOperationalConditions.pdf
